# Implementing the WHO Safe Childbirth Checklist modified for preterm birth: lessons learned and experiences from Kenya and Uganda

**DOI:** 10.1186/s12913-022-07650-x

**Published:** 2022-03-03

**Authors:** Kevin Abidha Achola, Darious Kajjo, Nicole Santos, Elizabeth Butrick, Christopher Otare, Paul Mubiri, Gertrude Namazzi, Rikita Merai, Phelgona Otieno, Peter Waiswa, Dilys Walker

**Affiliations:** 1 Kenya Medical Research Institute, Nairobi, Kenya; 2 Makerere University School of Public Health, Kampala, Uganda; 3 Institute for Global Health Sciences, University of California, San Francisco, San Francisco, USA; 4 Department of Global Public Health, Karolinska Institutet, Stockolm, Sweden; 5 Dept. of Obstetrics, Gynecology and Reproductive Sciences, University of California, San Francisco, San Francisco, USA

**Keywords:** Safe childbirth checklist, Preterm birth, Intrapartum care, Quality of care, Evidence-based practices

## Abstract

**Background:**

The WHO Safe Childbirth Checklist (SCC) contains 29 evidence-based practices (EBPs) across four pause points spanning admission to discharge. It has been shown to increase EBP uptake and has been tailored to specific contexts. However, little research has been conducted in East Africa on use of the SCC to improve intrapartum care, particularly for preterm birth despite its burden. We describe checklist adaptation, user acceptability, implementation and lessons learned.

**Methods:**

The East Africa Preterm Birth Initiative (PTBi EA) modified the SCC for use in 23 facilities in Western Kenya and Eastern Uganda as part of a cluster randomized controlled trial evaluating a package of facility-based interventions to improve preterm birth outcomes. The modified SCC (mSCC) for prematurity included: addition of a triage pause point before admission; focus on gestational age assessment, identification and management of preterm labour; and alignment with national guidelines. Following introduction, implementation lasted 24 and 34 months in Uganda and Kenya respectively and was supported through complementary mentoring and data strengthening at all sites. PRONTO® simulation training and quality improvement (QI) activities further supported mSCC use at intervention facilities only. A mixed methods approach, including checklist monitoring, provider surveys and in-depth interviews, was used in this analysis.

**Results:**

A total of 19,443 and 2229 checklists were assessed in Kenya and Uganda, respectively. In both countries, triage and admission pause points had the highest rates of completion. Kenya’s completion was greater than 70% for all pause points; Uganda ranged from 39 to 75%. Intervention facilities exposed to PRONTO and QI had higher completion rates than control sites. Provider perceptions cited clinical utility of the checklist, particularly when integrated into patient charts. However, some felt it repeated information in other documentation tools. Completion was hindered by workload and staffing issues.

**Conclusion:**

This study highlights the feasibility and importance of adaptation, iterative modification and complementary activities to reinforce SCC use. There are important opportunities to improve its clinical utility by the addition of prompts specific to the needs of different contexts. The trial assessing the PTBi EA intervention package was registered at ClinicalTrials.gov NCT03112018 Registered December 2016, retrospectively registered.

**Supplementary Information:**

The online version contains supplementary material available at 10.1186/s12913-022-07650-x.

## Background

Globally, 193,600 maternal deaths occur annually and 1.8 million children die in the first month of life [1]. The majority of these poor outcomes are in low- and middle-income countries (LMIC) with most deaths recorded in the intrapartum period. Most maternal deaths are attributed to direct obstetric causes; hemorrhage, hypertensive disorders and sepsis account for about 73% of these deaths [2]. Among newborns, preterm birth (28%), birth asphyxia (23%) and infection (36%) are the leading causes of death [3]. To end preventable maternal and newborn mortality and morbidity, the WHO notes every pregnant woman and newborn needs skilled care at birth [4].

Quality of care for maternal and newborn services in LMICs remains a challenge, despite existence of evidence-based practices (EBPs) [5]. To improve quality of care and avoid preventable deaths among mothers and newborns, the WHO introduced the Safe Childbirth Checklist (SCC) [6]. The SCC is a paper-based tool developed to support facility-based health workers to perform essential EBPs during the intrapartum and immediate postnatal periods, focusing on preeclampsia, post-partum hemorrhage, infection, obstructed labour, and birth asphyxia. The WHO has called for wider testing and improvement of the SCC in different environments to further investigate its implementation in varying contexts [7].

The checklist has been tested in countries across Latin America, Asia and Africa. Results from 38 research teams from 19 different countries show that the SCC is easy to use and improves quality of care [8]. A coaching-based WHO checklist quality improvement program in India showed increased adherence to essential birth practices—72.8% in the intervention versus 41.7% in the control group [9]. In another pre-post intervention study in India, it improved the number of EBPs used around the time of birth from 10 to 25 of 29 practices (*p* < 0.001) [10]. In Bangladesh, there was a 70% improvement in the delivery of EBPs with introduction of the SCC [11]. However, despite improved adherence to EBPs among providers in the intervention arm of the coaching-based India program, there was no difference in maternal and perinatal mortality or maternal morbidity [9]. Another study in Rajastan, India demonstrated that use of the checklist could avert up to 40,000 intrapartum deaths due to stillbirths [12].

Little research has been conducted in East Africa on use of the SCC to improve intrapartum care, particularly for preterm births despite the burden of prematurity. We implemented an intervention package including use of a modified safe childbirth checklist (mSCC) through the East Africa Preterm Birth Initiative (PTBi EA) to improve quality of care for preterm babies in Busoga Region, Uganda and Migori County, Kenya [13, 14]. For this cluster randomized controlled trial (cRCT), 10 control sites received data strengthening (DS) and the mSCC, while 10 intervention sites received DS, mSCC, with the addition of PRONTO simulation and team training, and quality improvement (QI) Collaboratives. The DS intervention included review of maternity registers and monthly reports for completeness and review of key indicators. The mSCC intervention included training and provision of a locally-adapted mSCC into maternity charts. PRONTO simulation and team training included bedside mentoring and regular review of clinical skills and teamwork in simulation activities. QI included facility-based teams performing Plan-Do-Study-Act (PDSA) cycles, with guidance from a study QI mentor and cross-facility collaborative learning sessions every 4–6 months. The intervention package was also introduced at three referral hospitals (two in Uganda and one in Kenya) which were not included in the cRCT primary analysis. The trial’s results are reported elsewhere; briefly, 347 (23%) of 1491 infants in the control group were stillborn or died in the neonatal period compared with 221 (15%) of 1447 infants in the intervention group (odds ratio 0·66, 95% CI 0·54–0·81) [14]. A description of the overall intervention package has been previously published [15], and further information about the package elements and Logic Model is provided in Additional File 1.

While it is not possible to disentangle the respective impact of the components of the intervention package, closer examination of each intervention is warranted given the trial’s positive results. The aim of this paper is to describe the adaptation, implementation and improvements for uptake of the PTBi EA mSCC, reflecting on country-specific experiences and lessons learned.

## Methods

### Study design

To assess the implementation and uptake of the mSCC across study sites, we employed a mixed-methods approach. Review of medical charts containing the mSCC were employed to quantitatively ascertain mSCC completion rates across study facilities, while surveys and interviews assessed provider perceptions of the mSCC.

### Intervention development and implementation

The mSCC was introduced across all 23 study facilities; 10 control, 10 intervention and 3 referral facilities. The checklist was integrated into the patient charts in maternity wards for all mothers who were admitted for labour and delivery. Healthcare workers used the checklist to remind themselves of EBPs that should be performed to ensure quality of care for mothers during labour, delivery and immediately post-delivery.

Facility stakeholders and research teams together undertook the process of modifying the SCC. Key adaptations included: (1) integration of a triage pause point upon presentation to the hospital for assessments done prior to admission; (2) an added focus on gestational age assessment and diagnosis and management of preterm labour; and (3) alignment with Kenya and Uganda national obstetric and neonatal care guidelines. The addition of the triage pause point focused on assessment of gestational age and indications for potential complications (e.g., high blood pressure, fever, bleeding) in order to assist providers in making the decision to admit, refer or send the woman home. If a woman was admitted, this triage information would also aid in subsequent care (e.g., provision of antenatal corticosteroids or antibiotics). For example, a woman in preterm labor could be sent home without being admitted because labor is not advanced enough and contractions may subside; however, she might benefit from admission for administration of antenatal corticosteroids if she is at risk for a preterm delivery.

Each country's mSCC was reviewed with key stakeholders including clinicians and facility leadership and was piloted before introduction. Pilot testing was done in March 2016 at one and two facilities in Kenya and Uganda respectively, with formal introduction of a revised version during DS activities from May to August 2016.

Ongoing use of the mSCC was supported through complementary mentoring at all sites alongside DS throughout the study period. In control sites, this mentoring included an initial training followed by several days of on-site training and direct coaching. Subsequently, study staff monitored checklist availability and uptake through completion monitoring and provided training for new staff or referesher trainings on request. Completion monitoring results were provided to facility staff.

In intervention facilities, PRONTO training and QI activities further supported mSCC use. The staff shared challenges faced with the checklist during feedback sessions, and gaps were addressed together by the PTBi EA study teams and the maternity staff. The mSCC was used during PRONTO trainings and simulation activities to reinforce use, and the EBPs included in the checklist were covered in the PRONTO curriculum and bedside mentoring. QI teams were encouraged to use data points from the checklist in their PDSA cycles, including uptake of key EBPs such as Kangaroo Care and antenatal corticosteroid use. Additionally, at Kenya intervention sites, the QI Collaborative tracked mSCC completion as a QI indicator.

In Kenya, in response to low uptake in the first 6 weeks after mSCC launch and upon urging by the county leadership, providers were incentivized (USD$0.50) for each checklist completed. Incentives were offered in both intervention and control sites, for all births irrespective of whether the infant was recruited for follow-up in the main study or not. In Uganda, financial incentives (paid quarterly to maternity ward in-charges) were given to staff related to cRCT enrollment and consent, but not linked to checklist completion.

The mSCC adaptation and implementation process for each country are summarized in Table 1. The final checklist for each country is provided in Additional Files 2 and 3. Additional File 4 includes a timeline of the implementation of the intervention package and the data collection activities for this analysis.

**Table 1:** Implementation approach by country

Stage	Uganda	Kenya
**Modification**	•Added triage pause point •Adapted content for preterm birth focus	•Added triage pause point •Adapted content for preterm birth focus
**Piloting**	•One-week pilot in two facilities	•One-week pilot with 6 providers in one facility
**Post-pilot Adjustments**	•Shortened checklist •Appended in patient chart	•Shortened checklist •Appended in patient chart
**Launch**	•Training of in-charges and facility-data staff •On-site training and direct coaching of providers in completion	•Training of in-charges and facility-data staff •On-site training and direct coaching of providers in completion
**Adjustments post-launch**	•Distributed time points through different sections of the patient chart	•Added a USD$0.50 incentive per completed checklist, distributed to maternity in-charges
**Monitoring**	•Monthly or bi-monthly convenience samples of charts checked for completion	•Census of charts reviewed for checklist completion among all charts •Individual-level data extraction for evidence-based practices among preterm eligible cases
**Reinforcement**	•Data team provided feedback, weekly for first month then monthly for 6 months •Completion results displayed on Data Dashboard •QI teams used mSCC as a data source (intervention sites only) •PRONTO training reinforced checklist use (intervention sites only) •As needed refresher trainings of the facility staff by study team	•Data/clinical team provided feedback in quarterly meetings •Completion results conveyed to in-charges •QI teams used mSCC as a data source (intervention sites only) •QI collaborative tracked mSCC completion as an indicator (intervention sites only) •PRONTO training reinforced checklist use (intervention sites only) •As needed refresher trainings of the facility by study team

Unless noted, activities were for all sites, inclusive of control, intervention and referral facilities

### Study setting

The mSCC was introduced using a regional approach in 17 facilities in Migori County, Kenya (including one referral hospital) and 6 facilities in the Busoga Region, Uganda (including two referral hospitals) [13, 14]. In Migori County, facility births represent 53% of all births and the 17 selected health facilities include approximately 10,000 deliveries annually [16]. In Busoga, approximately 77% of deliveries occur in facilities and the facilities included in this analysis have approximately 22,000 annual deliveries [17]. The sites consisted of 15 public and 2 not-for-profit missionary hospitals in Kenya, and 4 public and 2 not-for-profit missionary hospitals in Uganda.

Kenya sites were generally smaller (mean delivery volume 961 versus 3665 in Uganda), and included four Health Center (levels IIIs and IVs) in addition to sub-county hospitals. Only four facilities in Kenya (including the referral hospital) had capacity for cesarean section, whereas all facilities in Uganda had this capacity.

Kenya’s study activities occurred from June 2016 to April 2019. Kenya’s overall timeline was delayed due to a nurses’ strike between June 2017 and November 2017. In Uganda the study began in May 2016 and ended in May 2018.

### Participants and sampling

#### Completion monitoring

In Kenya, all checklists distributed to the 17 facilities were reviewed to assess uptake rates between January 2018 to March 2019 after all study facilities were re-opened post-strike. In Uganda, a convenience sample was drawn from each of the 6 study facilities between December 2016 to December 2017; 10% of patient charts from monthly deliveries were randomly selected and checked for checklist completeness.

#### Surveys

A process evaluation survey (Additional File 5) including inquiries about mSCC perceptions was developed and administered in Uganda (May 2018) and in Kenya (August 2018). In Kenya, surveys were administered to 5 healthcare workers across various cadres at 7 control and 7 intervention sites (total 70 surveys). Participants were selected among healthcare workers who worked in either maternity or newborn unit for more than 3 months. In Uganda, health workers who had participated in the cRCT were purposively sampled and identified by the facility in-charge at each site (total 118 surveys).

#### Interviews

Kenya’s process evaluation included in-depth interviews (IDIs) and focus group discussions (FGDs) among diverse PTBi EA stakeholders. However, since frontline workers were the most relevant to our goals of understanding perceptions toward mSCC use, we examined a subset of these—11 IDIs from 7 intervention and control facilities. The interview guide is available as Additional file 6. Health workers were purposively sampled if they worked in maternity, were familiar with the study, and were available when the site was visited by the mixed gender consultant team led by a professional degree holder. All interviewers had experience in qualitative research, were trained on the instruments, but had no prior relationship with the sites. In Uganda, 4 IDIs (Additional File 7) were conducted in July 2019 from each of the 4 study sites (16 interviews total) to capture perceptions, acceptability and sustainability of the mSCC across intervention and control facilities. For Uganda, these IDIs were completed post-cRCT and respondents were purposively sampled as those that had used the checklist, and IDIs were conducted among referral hospital providers, but not included in this study. Interviews were conducted by male Uganda study team members, including one of the authors (DK, a professional degree holder). All had prior experience in qualitative work, contributed to the development of the instruments, and were known to the participants as a staff member of the project. Interviewers in both countries approached potential respondents in person, after previously arranging with facility management to visit on an agreed upon day and asking for names of staff familiar with the project. Interviewers introduced themselves and presented the goals of the interview as understanding the conduct of the project and how it could be improved but did not share their personal views. There were no refusals or drop-outs.

### Data collection

#### Completion monitoring

In both countries, the checklist was filled out by healthcare workers, primarily nurses, midwives and some clinical officers/medical doctors. In Kenya, completion monitoring data were collected on all maternity charts from each facility between January 2018 and March 2019. To assess mSCC completeness in Uganda, the PTBi EA data team visited each of the 6 facilities during 9 visits between December 2016 to December 2017 and reviewed a convenience sample of 10% of monthly deliveries. Since data were collected at varying time periods, time points were generated based on visit number to the facility (1 = first visit to facility, 2 = second visit, etc.).

We categorized completeness of the checklist by pause point as fully completed, partially completed or blank (no section filled). Confirmation of the uptake of the mSCC in the facilities was accomplished by comparing the total number of admissions per month in the maternity register in each facility with number of mSCC used.

#### Surveys

Paper questionnaires were distributed to healthcare workers, completed anonymously, and collected at a subsequent visit in May 2018 in Uganda and in August 2018 in Kenya.

#### Interviews

In both countries, IDIs were conducted in English, using a guide developed by the study team in private locations and were audio-recorded after receiving participant’s consent. Interviews lasted 45–60 min. Data collection included one interviewer and one note taker. Data were collected to complete a pre-agreed number, without regards to or discussion of saturation.

### Data analysis

#### Completion monitoring

Quantitative data were cleaned and verified by the data teams and entered into customized Open Data Kit platform. Descriptive statistics were generated including frequencies and means using SPSS v25.02. Bivariate analyses included chi-square tests for categorical data and student’s t-tests for continuous data.

#### Surveys

In Kenya, survey data were entered into SPSS, checked and cleaned for possible erroneous outliers before descriptive statistics were generated. In Uganda, data were collected via paper forms and then entered into a Microsoft Access database, cleaned and verified. Descriptive analyses were conducted using Stata 15.1.

#### Interviews

Interviews were transcribed verbatim into Microsoft Word. A Framework Method [18] approach was used with a priori domains of interest related to perceived facilitators and barriers to implementation and potential for sustainability. Two independent coders reviewed all transcripts and coded recurring themes. A matrix was generated to synthesize themes and map relevant quotes. When the two coders did not agree or when the content was unclear, a third person served as an arbitrator. Participants were not involved in reviewing transcripts or results.

The COREQ checklist for qualitative work is included as Additional File 8 and the TIDieR checklist for reporting of implementation of the mSCC is included as Additional File 9.

### Ethical considerations

PTBi EA was granted ethical approvals from Higher Degrees, Research and Ethics Committee from Makerere University, KEMRI Scientific and Ethics Review Committee, and UCSF Committee on Human Research. Permission to extract non-identifiable aggregate data from medical charts, checklists and maternity registers was allowed under these approvals. Protocol was amended to include evaluation activities. Survey participants provided consent, using approved study consent procedures within the respective countries. Confidentiality was ensured by de-identification of the participants and restriction of access to the data to a small number of study staff.

## Results

### Completion monitoring

In Kenya, data were collected from a total of 19,443 checklists with an average of 1296 per month across facilities (SD ± 125). In Uganda, data were collected from a total of 2229 checklists with an average of 248 (SD ± 57) per visit.

**Fig. 1:**
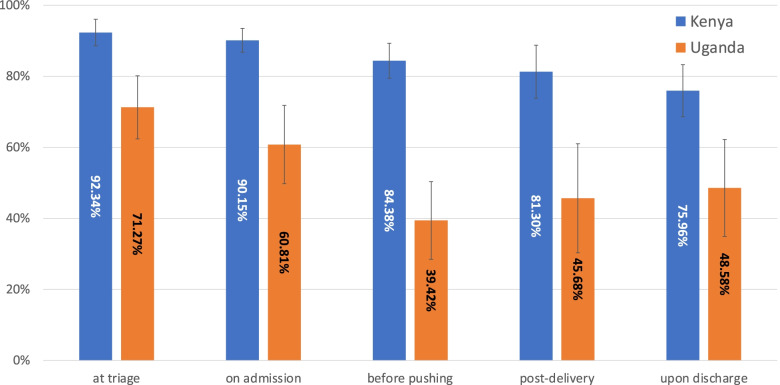
Average completion rates by pause point and by country across the mSCC monitoring period

Pause point-specific completion rates across monitoring time points in Kenya and Uganda were averaged to ascertain overall completion rates by pause point by country (Fig. 1). In both countries, the first two mSCC pause points—triage and admission – had higher rates of completion compared to the last three pause points – before pushing, post-delivery and discharge. This trend was more pronounced in Uganda than Kenya.

**Fig. 2:**
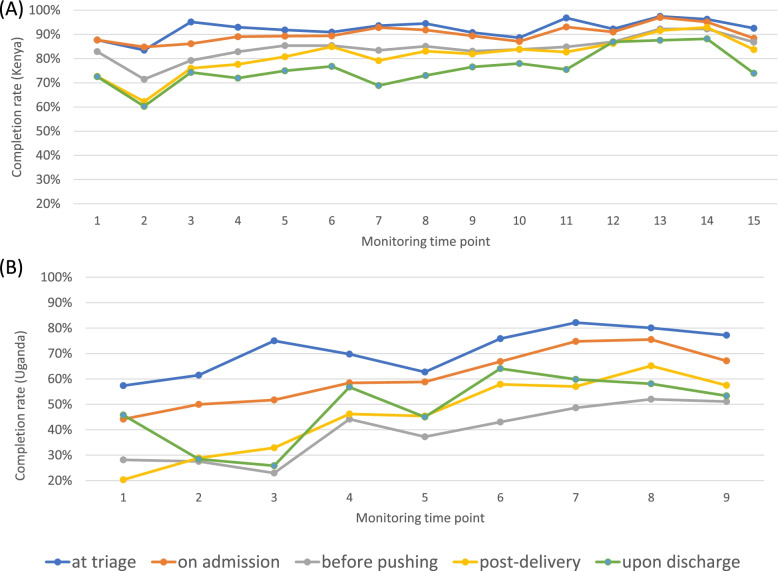
(**A**) Completion rates by pause point in Kenya over 15 time points (January 2018-March 2019); (**B**) Completion rates by pause point in Uganda over 9 time points (December 2016- December 2017)

To assess completion rate trends over time in each country, data were disaggregated by monitoring time point. In Kenya (Fig. 2A), completion across all facilities exceeded 70% for most time points. Across time points, the first two pause points were more consistently filled out. In Uganda, there was more variability (Fig. 2B) in pause point-specific uptake across facilities. Completion rates ranged from 80% (at triage) to 20% (post-delivery). Similar to Kenya, the first two pause points were more frequently completed. An increase in mSCC completion over the study duration was observed in Uganda.

To investigate if exposure to the PTBi EA package enhanced uptake, we analyzed completion rates by cRCT study arm (i.e., control versus intervention). Table 2 below shows higher rates of completion at each pause point in intervention facilities compared to control facilities. Additionally, 3 referral facilities were exposed to the package, but were not included in the cRCT’s primary analysis due to inability to pair-match them. These hospitals had an average annual delivery volume of 5672 deliveries (± 1073) and were larger in volume than the 20 cRCT sites. At these referral sites, completion of the checklist during all pause points was significantly lower than control or intervention facilities, except for the discharge pause point (Table 2).

**Table 2:** Completion rates by pause point in control vs. intervention facilities

Pause point	Control (*n* = 143)	Intervention (*n* = 129)	Referral (*n* = 33)	*P *value (ctrl vs. intervention)	*P *value (ctrl vs. referral)	*P *value (intervention vs. referral)
At triage	87.12	94.45	72.28	< 0.001	< 0.001	< 0.001
On admission	83.68	92.43	61.59	< 0.001	< 0.001	< 0.001
Before pushing	74.37	86.93	44.33	< 0.001	< 0.001	< 0.001
Post-delivery	72.54	86.26	41.89	< 0.001	< 0.001	< 0.001
Discharge	63.48	81.42	64.48	< 0.001	0.820	< 0.001

### Provider perceptions (surveys)

A total of 69 and 118 providers completed the process evaluation survey in Kenya and Uganda, respectively (Table 3). Most respondents were nurses or midwives. Respondents in Uganda had more years of clinical experience compared to their Kenyan counterparts. Respondents from private not for profit facilities made up only 9% of the sample, so results were not broken down by facility type.

**Table 3:** Characteristics of mSCC questionnaire respondents

Characteristics	KENYA		UGANDA	
	***N* = 69**	**(%)**	***N* = 118**	**(%)**
**Location of service**				
Referral Hospitals	0	0	51	43.2
Other Public Hospitals	61	88.4	58	49.2
Mission Hospitals	8	11.6	9	7.6
**Provider type**				
Nurse	32	46.4	13	11.0
Midwives	17	24.6	96	81.4
Clinical Officer (CO)	14	20.3	1	0.8
Physician/medical officer	2	2.9	4	3.4
Nursing assistant	1	1.4	0	0.0
Student/intern	1	1.4	0	0.0
Other	0	0.0	2	1.7
N/A	2	2.9	2	1.7
**Years of service**				
< 5 years	25	36.2	32	27.1
5–10 years	25	36.2	38	32.2
11–20 years	13	18.8	31	26.3
> 20 years	6	8.7	17	14.4

Table 4 provides respondents’ perceptions regarding the mSCC mid-study, by study arm. In both countries, most strongly agreed that the mSCC was easy to read and follow and helped in clinical decision-making, particularly for preterm labor, preeclampsia, and maternal infection. Responses by providers in intervention sites were more favorable than those in control sites in both countries, and were overall slightly more favorable in Uganda Perceived clinical utility regarding diagnosis and management of multiple gestation and referrals differed among respondents, again with more favorable responses in Uganda intervention sites, but less favorable responses in Uganda control sites compared to Kenya. However, respondents also felt that the mSCC repeated information in the patient chart and maternity register, particularly in Uganda. In Kenya, almost one-third of respondents believed the mSCC made their job more difficult.

**Table 4:** Provider perceptions regarding mSCC and clinical utility

	KENYA *n* = 69				UGANDA *n* = 118			
	Control *n* = 34 Strongly Agree		Intervention *n* = 35 Strongly Agree		Control *n* = 25^a^ Strongly Agree		Intervention *n* = 75 Strongly Agree	
**The mSCC:**	n	%^b^	n	%	n	%	n	%
**Was easy to read**	17	52	25	74	11	44	57	76
**Was easy to follow**	20	63	26	76	17	68	65	87
**Makes job easier**	17	53	26	76	16	64	62	83
**Repeats information in patient chart**	7	21	9	27	10	40	32	43
**Repeats information in maternity register**	4	13	10	31	9	36	28	37
**Helped in clinical decision making**	18	56	28	82	16	64	63	84
**The mSCC helped in diagnosis and management of:**								
**Preterm labor**	24	75	26	76	16	64	62	83
**Preeclampsia**	19	63	25	74	14	56	60	80
**Multiple gestation**	14	47	15	44	14	56	42	56
**Maternal infection**	15	48	21	64	15	60	60	80
**Referrals**	10	30	18	56	2	8	37	49

^a^Missingness: In Kenya missingness varied by question from 2–9%. In Uganda, 15% of surveys were excluded for high rates of missingness

^b^Percentages reflect the percentage among respondents who answered the question

Regarding incentives in Kenya, 82% in the intervention and 73% in the control sites stated that they would continue filling the checklist even without the financial incentives, whereas 6% and 15% of respondents would not.

### Provider perceptions (interviews)

The qualitative component of this study explored providers’ perceptions and experience with the mSCC either mid-study (Kenya) or post-study completion (Uganda). Of the 11 interviews in Kenya, 3 were with men (2 Nursing officers and a Clinical Officer) and 8 with women (4 Nursing Officers, 3 Maternity In-Charges and 1 Maternity Nurse). Of the 16 interviews conducted in Uganda, all were with women (13 Midwives, 2 Nursing Officers, and 1Medical Officer. Data are presented under four thematic areas: (1) factors influencing ease/difficulty of use; (2) influence on clinical decision-making by pause point; (3) perceptions toward implementation process; and (4) recommendations for improvement/potential sustainability. Responses did not vary notably by facility type.

#### Theme #1: factors influencing ease/difficulty of use

Several respondents noted that the mSCC sometimes served as a standalone tool when other standard documents were not available, thus increasing its uptake. Specifically, it replaced patient charts that were lacking in the facilities (Kenya) or exercise books that mothers brought with them to the maternity ward (Uganda).

*Sometime back we ran short of files so, it was the only document which we could produce to monitor a mother in labour. (Kenya, Nursing Officer, Intervention site)*

*Before Makerere came on board, we used exercise books that are just plain have no order like this file... Also, because the file can [more] easily be kept than the book that can be torn, the file is in order. (Uganda, Nursing Officer, Control site)*

Across both countries, the mSCC was initially regarded as an additional burden, largely because of its length. Competing workload and responsibilities, as well as onboarding of new providers/staff, were identified as barriers to uptake.

*We used to have few staff and maybe we have five deliveries in a day. … but again when you have many mothers and you are alone sometimes most staff tend to miss filling them. So, despite the fact that it is informative most staff fail to fill them especially when we have high workload. (Kenya, Clinical Officer, Intervention site)*

*…Actually, some people were not interested, others did not know how to use it and could not fill some parts. Also, workload and you are alone, the mother comes in second stage, so by the time you come and fill in the checklist, you can’t remember especially when they are many. (Uganda, Midwife, Control site)*

However, by study completion in Uganda, clinical utility of the mSCC became clear as providers became more familiar with its content and flow. Eventually, despite its length, the checklist’s value was appreciated by many, thus encouraging uptake.

*When I saw this checklist and it seemed long in terms of the content… but yet it used to guide; someone may not know for example the dosage of dexamethasone or what is supposed to be given to a preterm labour and she is 28 weeks, it served as a guideline. There are certain things that are within that checklist that I personally found useful during my work, so that was the benefit, the disadvantage was the volume, but we got used [to it]. (Uganda, Medical officer, Control site)*

*Because it was new and you take a lot of time asking the mothers, but now that I am used to it, I don’t even want to leave it. We are now used to the checklist. Given that it is filled at different times and probably by different people, it is not that tasking to fill. It became easy to fill… That was at the beginning, every bit is important, after all its just for ticking. (Uganda, Midwife, Intervention site)*

#### Theme #2: influence on clinical decision-making by pause point

Specific comments were made about the value of the triage and admission pause points in that it aided in recognition of preterm labour, whether a doctor was needed for complicated cases, and preparation for a potentially complicated case.

*What I like about is that it helps a midwife to capture the key areas that you need to know like the LMP, gestation period, because our interest is to get a healthy living and safe delivery so it helps you to know the key areas you need to check. (Kenya, Maternity In-charge, Intervention site)*

*If a mother is admitted with preterm labour, I am able to make early preparations to administer dexamethasone, preparing the mother for any complications like respiratory distress syndrome, so basically it helps us to ensure that the mother and the baby survive after assessing her on admission. (Uganda, Midwife, Control site)*

*It was good because when a patient comes, you are able to know who to deal with this patient either a case for the midwife or the doctor, you separate patients for doctors to handle and for the midwife to handle… like preeclampsia, we are supposed to give first aid but then call a doctor to manage… (Uganda, Midwife, Control site)*

While these early pause points were highlighted as useful, the discharge pause point was mentioned as a challenge specifically in Uganda, especially due to workload.

*At discharge, that one was a bit challenging… It’s a short part that can be easily filled but maybe there was laziness among the workers. At discharge I think the midwives would see that may be the patient is now fine so it could be hard for them to go back in the checklist... (Uganda, Midwife, Intervention site)*

*Now the challenging [sections] - after delivery when a mother has not yet delivered, midwives tend to be on some kind of tension… I have realized that after delivery the postnatal there, someone thinks everything is over so those sheets after the delivery notes like at discharge, or before discharge, after delivery it has been a challenge and you find that all these ones before delivery are filled. (Uganda, Principal Nursing Officer, Intervention site)*

#### Theme #3: perception toward implementation process

Across both countries, integration of the mSCC into medical charts helped uptake significantly. This allowed providers to use the chart in order of provision of care and helped with care continuity for each client even considering provider handoff and competing responsibilities.

*They helped us by making files and incorporating it into the patient charts, it was triage, admission, before and after delivery and at discharge. So, at the introduction they called us and we were oriented to the checklist and they asked us if they are friendly. In the beginning we had issues that it was big to fill but as we went along with it, it was user friendly. If you just work without that file you can forget so many things but when you have this file, you can do so many things…(Uganda, Midwife, Intervention site)*

*We make sure that every file has a checklist. So, it’s attached to the files… Once it is put in the files we realize that it has become easier. (Kenya, Health Facility In-charge, Control site)*

*We had agreed that when someone is doing their handing over, we look at the checklist so that we ensure they are done well by anyone on duty at a particular time. (Kenya, Clinical Officer, Intervention site)*

Continuity of mentorship, particularly through DS (all sites), PRONTO and QI activities (intervention sites) were cited as mechanisms of reinforcement and recognition of utility.

*They [PRONTO mentors] usually go through the checklist and see what happened if there were deliveries that were conducted in a week then they tend to study them and see where there is a gap so when there is mentorships they are doing mentorships based on their findings of all the complications. (Kenya, Clinical Officer, Intervention site)*

*The mentors did a great job because … change is not easy and I remember in the beginning I heard that the interest of the midwives with these new things was not there but they kept on coming and encouraging people, … so that kind of persistence and the love to help people do something. I really appreciate because it took a long time especially with [hospital] to adapt this tool. (Uganda, Principal Nursing Officer, Intervention site)*

In Kenya specifically, inclusion of mSCC completion as a QI indicator was also a catalyst for uptake at intervention facilities, while incentives were also referenced.

*Yeah, they [PTBi team] always collect data through the checklist, but as the facility we always collect that data before they come so that we can work on it as the work improvement [QI team] team, so that we see what goes on…so we can really maintain that. With this it really shows us how we are moving on …(Kenya, Nursing Officer In-charge, Intervention site)*

*They were not filling it until PTBi came with some incentives that if you fill it rightly, correctly and completely filled and up to the end… they will give 50shillings per rightly filled checklist from there people picked and up to date they are filling checklist (Kenya, Maternity In-charge, Intervention site)*

#### Theme #4: recommendations for improvement/potential sustainability

When asked how the mSCC could be improved for future uptake, some respondents wanted shorter checklists. At the same time, some felt that it should be expanded to include more space for clinical notes – underscoring how this tool was often used as a documentation tool.

*It is very useful but, is there a way in which it can be compressed more so that we have less questions but all are covered so that we don’t repeat all the question all the time…. (Kenya, Nursing Officer, Intervention site)*

*The most recent version we got is better, it has been reduced. We also need additional space for Doctor’s notes and the observations for mothers that take long. (Uganda, Midwife, Control site)*

There was a clear desire from many respondents that they hoped the mSCC would continue beyond the life of the PTBi project. Many recognized, however, the role of Ministry or facility administration in making it sustainable.

*The one to continue is the checklist… I think we need to make it continue and put to task the county for the sustainability. We know sustainability may not be able to continue, so we need when the county is printing maternity file then there should be a sustainability. (Kenya, Nursing Officer In-charge, Intervention site)*

*What I can comment about its availability - the hospital needs to have an active administrative aim so that they are able to make photocopies and we attach them in the files because we make the files locally. And I think it’s possible, surely its possible, because we do a lot of photocopies for other files so adding this tool for as long as it’s helpful. (Uganda, Principal Nursing Officer, Intervention site)*

## Discussion

We explored country-specific experiences and provider perceptions regarding implementation strategies and checklist utility, and how these might influence uptake rates across different types of facilities. In both countries, there was an overall improvement in the use of the checklist over time, suggesting perceived clinical utility and workflow integration can amplify uptake. We also found higher uptake and improved perception of the mSCC in intervention facilities compared to control facilities. This was attributable perhaps to reinforcement of its use by the other 2 components of PTBi EA intervention package, i.e. PRONTO and QI activities.

The mSCC was perceived to be clinically useful. We showed that triage assessment and prompts at admission were useful, as exhibited by higher uptake at these pause points and reinforcing qualitative data. Notably, according to survey data, Ugandan providers tended to rate clinical utility higher than Kenyan providers, for conditions such as pre-eclampsia, preterm labour and maternal infection. This could be due to the fact that Uganda facilities were district level hospitals and higher, all of which offered Cesarean capacity. On the other hand, the 17 Kenya facilities included health centers level III or IVs, hospitals and the county referral facility; only four had Caesarean capacity. The level of clinical baseline experience and knowledge may differ by type of facility. Additionally, in Kenya, most maternity ward providers were nurses, while in Uganda, many of the nurses had additional midwifery training. Similarly, among survey participants, more Ugandan providers had > 10 years of experience as compared to Kenyan respondents.

Iterative adaptation of implementation strategies and inclusion of continuous reinforcing activities enhanced uptake. In our PTBi EA experience, integration into existing medical charts to align with workflow bolstered uptake, and ongoing mentorship through DS, PRONTO and QI helped enhance its use. The latter point is clear in that intervention facilities had higher rates of completion by pause point compared to control facilities. Similarly, our survey results showed that providers in intervention sites had more favorable perceptions of the mSCC. The value of complementary activities was previously well-highlighted in the BetterBirth program, where they included peer-to-peer coaching, leadership engagement and data feedback loops [19]. In a smaller study at a district hospital in Namibia where they saw an increase in EBPs from 68 to 95% and reduced perinatal mortality (22 to 13.8/1000 deliveries) over a 6-month period, the SCC was implemented under the guidance of a QI team [20]. The authors concluded that local leadership and continuous coaching through PDSA/QI activities enhanced acceptability. In contrast, however, in two tertiary high-volume hospitals in Sri Lanka (4000 and 9000 annual births), they demonstrated low checklist adoption rates of 54.3% and 18.8% [21]. The authors attributed poor uptake to lack of staff, inadequate training, short duration of implementation, and lack of institutional involvement. This hands-off approach, where the SCC was not implemented alongside mentorship, shows the importance of careful introduction and planned reinforcement.

Incentives may have been effective for short-term implementation, but provider-driven recognition of clinical utility may have enhanced use more organically. Kenya had consistently higher completion rates across monitoring time points, suggesting the incentive spurred uptake, although a high proportion of Kenyan providers stated they would continue the checklist without incentive mid-study. However, in Uganda, while some financial incentives were given to staff related to the study overall, they were not linked to checklist completion. There, we observed a gradual increase in uptake in the mSCC at all pause points over time, possibly reflecting an increase in use as providers recognized the clinical utility.

Nonetheless, mSCC use was affected by workload. The checklist was perceived as an added task by some providers, and many individuals noted the need to address repetitive content. It was stated that completion of the checklist did not take much time once one is familiar with it. However, providers often perceived it as a burden at first and if they lack the time, training and support to master its use, they may never get beyond the point of thinking it adds to their workload. Workload, particularly with high patient volume, has been previously observed in studies in Burkina Faso, Cote d’Ivoire and Sri Lanka [22, 23]. Low rates of completion at the PTBi EA referral hospitals, which had the highest delivery volumes, underscores this critical implementation barrier. Additionally, in our study, staff attrition was high and staff rotations occurred at regular intervals, approximately every 6 months in Kenya and approximately every 2 years in Uganda. Thus, it is possible that our sampling of maternity records was adversely affected by fewer staff trained in mSCC use. Interestingly, however, IDIs revealed that the mSCC was adopted as a teaching tool in some hospitals, both for onboarding new staff and rotating students. Future implementation efforts should consider integration into continuous training opportunities, such as pre-service education or continuous medical education/continuous professional development sessions that may already exist in facilities.

Modification of the checklist to address identification and management of prematurity and tailoring to the local context, and adopting local suggestions such as integration into medical charts or incentive use in Kenya were necessary components of our work. Such local tailoring has been highlighted in various studies. For example, for the national adaption of the SCC in Columbia, the checklist was modified to include a focus on antihypertensives, as well as maternal treatment of syphilis given the high prevalence of this disease regionally [24]. At a Namibia district hospital, the SCC was modified to include identification and guidance of referrals, provision of ART for prevention of mother-to-child transmission of HIV, and use of antibiotics for non-facility-based births [25]. We similarly added prompts around provision of antiretrovirals and anti-malarials, given their relevance to preterm birth. At all pause points except just before pushing, we integrated prompts around gestational age determination, use of antenatal corticosteroids, and danger signs that might warrant referral for either mother or baby. A recent publication called for additions to the SCC to guide newborn care, where authors state the current WHO SCC lacks several key EBPs (e.g. cord care) and that prompts related to skin-to-skin contact and breastfeeding are inadequate [26]. PTBi EA stakeholders had similar sentiments given our focus on prematurity. Thus, specific newborn-related items were added, aligned with national guidelines. However, despite these PTBi EA adaptations, some gaps remain. For example, survey respondents felt that the mSCC had limited utility for identification of referral needs, highlighting how further improvement to the SCC, particularly around referral strengthening, is needed.

Sustainability outside of external funders and research activities remains a key issue. Many respondents across both countries called upon facility administration or the Ministry of Health to help sustain printing of the checklist after the cRCT ended. This call for sustainability was also compounded by the dearth of patient charts to begin with, as evidenced by how the mSCC was often used as standalone documentation. While we were gratified to see the checklist so valued, we would emphasize the importance of clinical charts first as they are better suited to ensure appropriate patient monitoring and handover with sufficient detail to support all aspects of quality of care. The need for strategic engagement with stakeholders early and throughout implementation has been previously acknowledged regarding potential scale-up of the SCC following studies in India [27]. Another threat to sustainability was the use of financial incentives to promote use of the checklist. However, despite knowing that such incentives will not be sustained, they were useful in overcoming initial resistance, as was seen in Kenya where most providers reported they would continue use even without an incentive.

### Limitations

There are a few key limitations to this study. Although the cRCT showed positive impact on health outcomes [15], it is not possible to determine the individual impact of the interventions that comprised the package, including the mSCC. It would have been ideal to obtain qualitative interviews mid-study and post-study across the two countries in order to better capture how perceptions evolved over time. In addition, purposive sampling of health providers who were familiar with the study may have introduced positive bias, which we attempted to mitigate by asking about challenges as well as successes. Although we had both public sector and private not for profit health workers included, there were not enough of the latter to allow for comparisons. For completion monitoring, differences in sample size between the two countries is considerable. In Uganda, the facilities were larger, and the sampling approach was determined by study resources, but felt to be sufficient for monitoring. In Kenya, a larger sample size was available due to the more intensive monitoring necessary as monetary incentives were linked to checklist completion. The timing of monitoring also differed; Kenya’s data reflects post-strike monitoring, well into the study period, while Uganda’s monitoring period was more representative of the study period. Nonetheless, the duration of the PTBi cRCT in both countries allowed us to observe trends over time which is a strength of this work. Lastly, the monitoring data is a retrospective analysis, rather than a reflection of actual use. Observational studies of the mSCC in action may enhance identification of opportunities for improvement. Regardless of these limitations, we used both quantitative and qualitative approaches to better understand mSCC implementation experiences across two countries among heterogeneous types of facilities.

## Conclusion

As the SCC continues to be implemented globally, this study sheds light on the importance of adaptation to local settings and creation of a supporting environment to reinforce its use. We highlight important opportunities to improve its clinical utility, including the introduction of a triage pause point and the addition of clinical prompts specific to the needs of each setting. Further research to enhance uptake post-delivery may be warranted.

## Supplementary Information

**Additional file 1.** The PTBi East Africa Intrapartum Package Includes additional information about the full PTBi intervention package and the logic model for the package

**Additional file 2.** Final mSCC Kenya Includes the final version of the modified Safe Childbirth Checklist used in the Kenya study sites

**Additional file 3.** Final mSCC Uganda Includes the final version of the modified Safe Childbirth Checklist used in the Uganda study sites

**Additional file 4.** Study Timeline Includes a timeline which shows implementation of all interventions as well as data collection activities for this analysis

**Additional file 5.** HW mSCC Feedback Survey Tool Includes the survey developed and administered in both countries to elicit feedback about the modified Safe Childbirth Checklist

**Additional file 6.** Kenya HW Interview Guides (English) Ver 1.0 March 2018 Includes the interview guide used in Kenya for healthworker interviews

**Additional file 7.** Uganda_IDI guide for post-implementation mSCC and Qi data collection Includes the interview guide used in Uganda for healthworker interviews

**Additional file 8.** ISSM_COREQ_Checklist_PTBI_mSCC Includes the completed COREQ checklist for the qualitative elements of this study

**Additional file 9.** TIDieR Checklist mSCC Includes the completed TIDieR checklist for the implementation of the mSCC described in this manuscript

## Data Availability

The datasets used and/or analyzed during the current study are available from the corresponding author on reasonable request.

## Abbreviations

WHO: World Health Organization
LMIC: Low- and middle-income countries
PTBi EA/PTBi: East Africa Preterm Birth Initiative
mSCC: Modified Safe Childbirth Checklist
EBPs: Evidence-based practices
DS: Data strengthening
QI: Quality improvement
CRCT: Cluster randomized controlled trial
IDI: In-depth interview
