# Supplementary material for: Implementing the WHO Safe Childbirth Checklist modified for preterm birth: lessons learned and experiences from Kenya and Uganda
Source: BMC Health Serv Res. 2022 Mar 3;22:294. doi: 10.1186/s12913-022-07650-x (PMC8896298; doi:10.1186/s12913-022-07650-x)
Supplement: Supplementary file 1 — Additional file 1. The PTBi East Africa Intrapartum Package Includes additional information about the full PTBi intervention package and the logic model for the package [file 12913_2022_7650_MOESM1_ESM.docx]

**The PTBi East Africa Intrapartum Package**

The PTBi East Africa study in Kenya and Uganda tested the impact of a package of interventions designed to improve quality of care. Two interventions – Data Strengthening and the Modified Safe Childbirth Checklist – were implemented in all sites, control and intervention. Two additional interventions – Quality Improvement Collaboratives and PRONTO simulation and team training – were implemented only in intervention facilities. The interventions, described in further detail below in a figure originally published in *The Lancet Global Health* with the full trial outcomes, worked synergistically together to reinforce evidence-based practices for mothers and newborns.


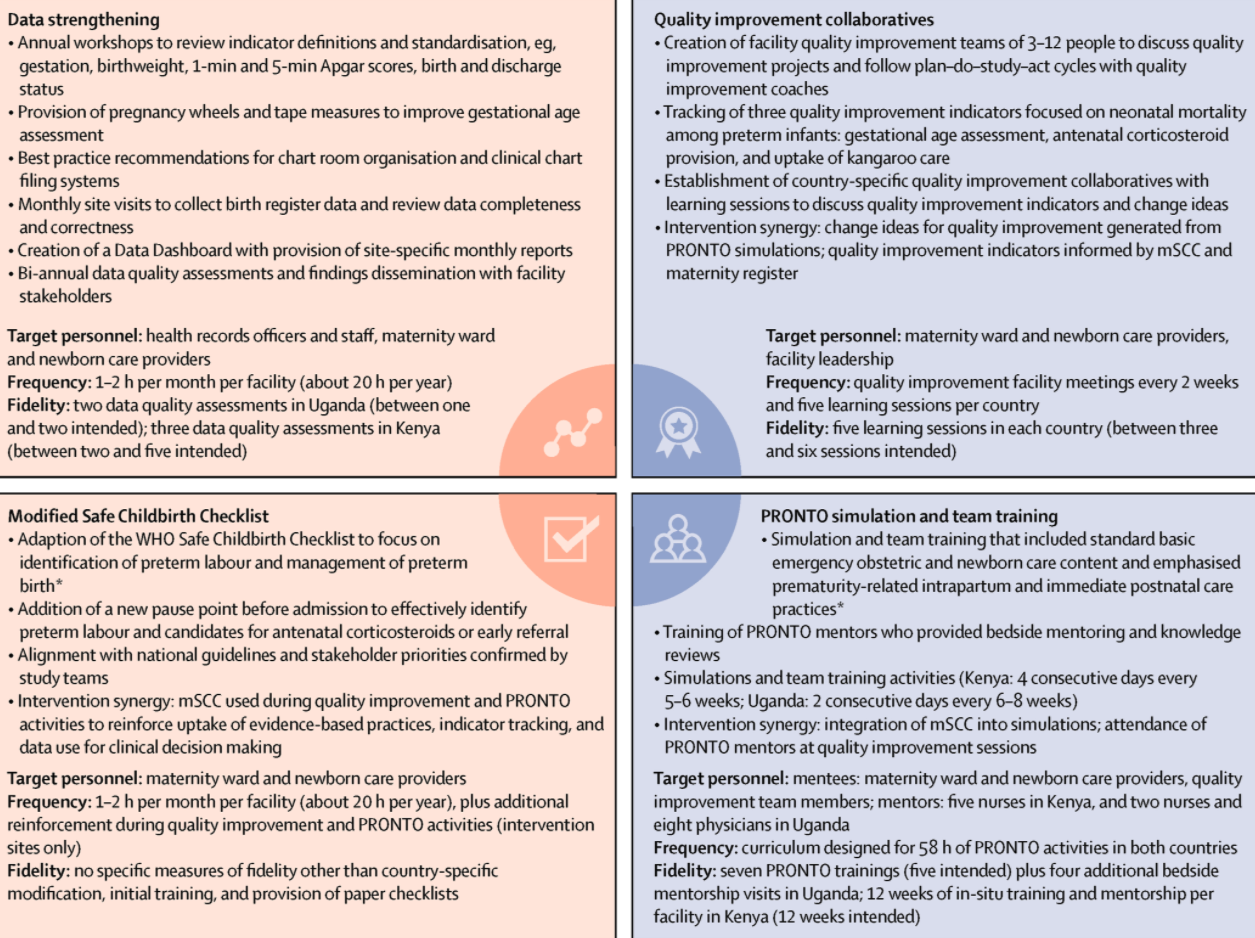


Figure originally published in: Walker D, Otieno P, Butrick E, Namazzi G, Achola K, Merai R, Otare C, Mubiri P, Ghosh R, Santos N, Miller L, Sloan N, Waiwa P. Effect of a quality improvement package for intrapartum and immediate newborn care on fresh stillbirth and neonatal mortality among preterm and low-birthweight babies in Kenya and Uganda: a cluster-randomised facility-based trial. *The Lancet Global Health* Volume 8 Issue 8 Pages e1061-e1070 (August 2020)

DOI: 10.1016/S2214-109X(20)30232-1

The study’s Logic Model, previously published with the trial protocol in *Trials*, shows how each of the interventions contributes to short-term and intermediate outcomes on the pathway to improved health outcomes.


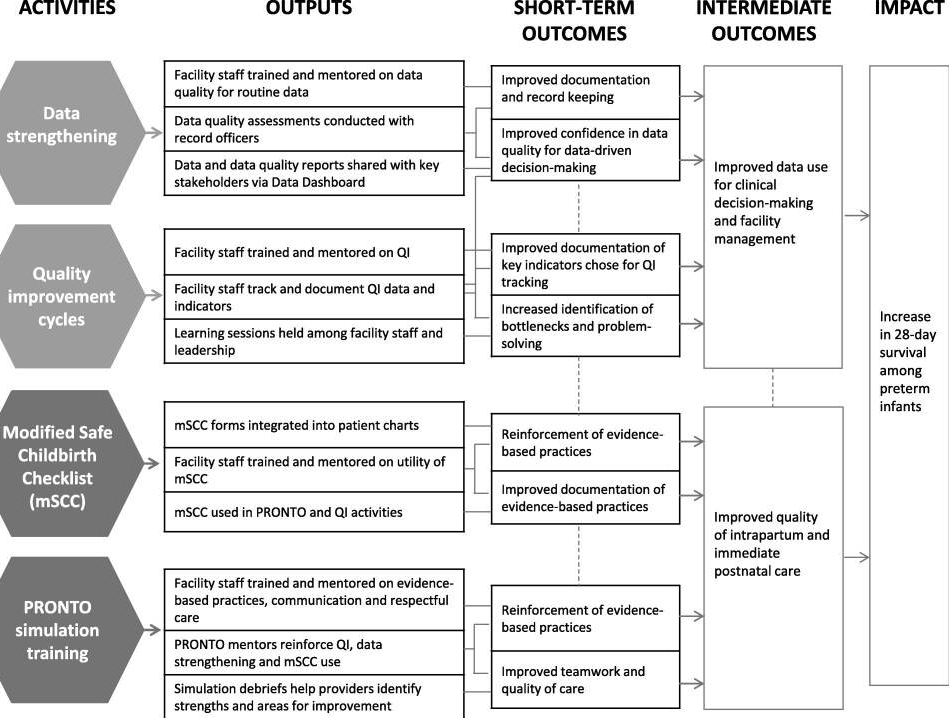


Figure originally published in: Otieno P, Waiswa P, Butrick E, Namazzi G, Achola K, Santos N, Keating R, Lester F, Walker D. Strengthening intrapartum and immediate newborn care to reduce morbidity and mortality of preterm infants born in health facilities in Migori County, Kenya and Busoga Region, Uganda: a study protocol for a randomized controlled trial. *Trials.* 2018 Jun 5;19(1):313. doi: 10.1186/s13063-018-2696-2. PMID: 29871696; PMCID: PMC5989441.
