# Supplementary material for: Implementing the WHO Safe Childbirth Checklist modified for preterm birth: lessons learned and experiences from Kenya and Uganda
Source: BMC Health Serv Res. 2022 Mar 3;22:294. doi: 10.1186/s12913-022-07650-x (PMC8896298; doi:10.1186/s12913-022-07650-x)
Supplement: Supplementary file 2 — Additional file 2. Final mSCC Kenya Includes the final version of the modified Safe Childbirth Checklist used in the Kenya study sites [file 12913_2022_7650_MOESM2_ESM.pdf]

# PRETERM BIRTH INITIATIVE - KENYA SAFE CHILDBIRTH CHECKLIST: FACILITY:______________________

| | | |
|---|---|---|
| **1** | **FIRST PRESENTATION OF THE MOTHER :** | |
| **Date:** | **Time:** | **Collected by:** |
| **Name:** | **DOB/Age:** | **File #** |
| **Gravidity:** | **Parity:** | **Phone number:** |
| **Sub-County** | **Village:** | |

| | | |
|---|---|---|
| 1. | *Record all that apply.*<br>Last normal menstrual period (LNMP): ____________<br>Gestational age (GA) by LNMP: __________________<br>GA by fundal height with tape measure, cm: _______<br>GA by ultrasound: _______________________________ | *If <37 weeks (preterm), admit or refer.* |
| 2. | Does patient have a history of preterm delivery?<br>□ Yes<br>□ No | *If yes, risk of preterm delivery is elevated.* |
| 3. | Does the mother need to be admitted for:<br>□ High blood pressure<br>□ High temperature/signs of active infection<br>□ Vaginal bleeding<br>□ High or low fetal heart rate<br>□ Preterm labor<br>□ Active Labor<br>□ Premature Rupture of membranes<br>□ Other, specify: _____________________________<br>□ NONE | ***Assess for indication that mother should be admitted or referred:***<br>• ***If systolic blood pressure >140 or diastolic blood pressure >90, admit or refer. Start management of pre-eclampsia.***<br>• ***If Temp >38°C or > 37.5°C axillary with signs of infection, admit or refer.***<br>• ***If FHR>160 or <110, admit or refer. Further monitor.***<br>• ***If preterm, initiate appropriate care or refer.***<br>• ***If dilation more than 4 cm, admit or refer.*** |
| 4. | Decision at this stage:<br>□ Admitted<br>□ Referred: Specify referral location: ____________________<br>□ Sent home: Indicate key plan/advise: ___________________<br>_______________________________________________________ | ***If unable to manage, refer.*** |

| | | |
|---|---|---|
| **2** | **ON ADMISSION TO MATERNITY** | |
| **Date:** | **Time:** | **Collected by:** |
| **Mother's Name:** | **Medical record/file #:** | |

| | | |
|---|---|---|
| 1. | Is baby likely to be born ≤34 weeks?<br>□ Yes<br>□ No | ***If YES, fill out <34 weeks section below.***<br>***If NO, skip to ALL MOTHERS section below.*** |
| | **IF MOTHER IS LIKELY TO DELIVER ≤ 34 WEEKS, CONSIDER CORTICOSTERIODS & TOCOLYTICS** | |
| 2. | Is she a candidate for Antenatal corticosteroids?<br>□ Yes<br>□ No | *Give antenatal corticosteroids per national protocol i.e. if no evidence of maternal infection and delivery is imminent within 24 hours to seven days*<br>**Dexamethasone 6 mg IM BD x 2/7 OR**<br>**Betamethasone 12 mg IM OD x 2/7** |
| 3. | Is she a candidate for tocolytics?<br>□ Yes<br>□ No | *Consider tocolytics (e.g. nifedipene) if giving steroids*<br>**Nifedipine 20 mg PO then 10-20 mg PO 4-8 hourly x 1-2/7** |
| 4. | Is she a candidate for Magnesium Sulfate for fetal Neuroprotection?<br>□ Yes<br>□ No | *Give for fetal neuroprotection if baby expected to be delivered before 32 weeks and delivery is imminent, within the next 24 hours*<br>**Magnesium sulfate 4 g IV with 5 g IM per buttock as initial dose (10 mg IM total), followed by 5 g IM q4 hours until delivery** |

| | | |
|---|---|---|
| 5. | Preparation for preterm delivery?<br>□ Yes<br>□ No | *Prepare for preterm birth*<br>• *Appropriate preterm bag and mask and/or PPV are available*<br>• *Continuous warmth for unstable preterms (e.g blankets)*<br>• *Ready to implement Kangaroo Care*<br>• *Ready to do delayed cord clamping (DCC)*<br>• *Staff assistance for delivery and resuscitation* |
| | **ALL MOTHERS** | |
| 6. | Confirm<br>□ Supplies ready to clean hands<br>□ Sterile gloves available for each vaginal exam?<br>□ Birth companion encouraged to be present at birth<br>□ Mother and companion informed to call for help if needed | *Call for help if any of the following:*<br>• *Bleeding*<br>• *Severe abdominal pain*<br>• *Severe headache or visual disturbance*<br>• *Unable to urinate*<br>• *Urge to push*<br>• *Convulsions*<br>• *Any other abnormality* |
| 7. | Does the mother require antibiotics?<br>□ Yes<br>□ No | *Give antibiotics if any of the following:*<br>• *PPROM*<br>• *Signs of infection (e.g Temperature ≥ 38°C, Foul-smelling vaginal discharge)*<br>• *Planned c-section*<br>• *Rupture of membranes > 18 hours* |
| 8. | Does the mother require Anti-malarials?<br>□ Yes<br>□ No | *Give anti-malarials if mother has:*<br>• *positive malaria smear*<br>• *meets other diagnostic criteria for malaria* |
| 9. | Does the mother require Antiretroviral medicines?<br>□ Yes<br>□ No | *If mother is HIV+, manage as per the guidelines* |
| 10. | Does the mother require Anti- hypertensive treatment?<br>□ Yes<br>□ No | *If systolic blood pressure ≥160 or diastolic blood pressure ≥110, or if approaching these levels.*<br><br>**For acute IV management:**<br>**Hydralazine 5-10 mg IV, if still elevated after 20 min, repeat dose OR**<br>**Labetalol 20 mg IV fist dose, if still elevated after 20 min, 40 mg Then initiate oral medication.** |
| 11. | Does the mother require Magnesium sulfate for pre-eclampsia?<br>□ Yes<br>□ No | *Give Mg $SO_4$ if patient has the following:*<br>• *systolic blood pressure ≥ 140 mmHg*<br>• *diastolic blood pressure ≥ 90 mmHg*<br>**AND** *any of the following:*<br>• *convulsions or coma*<br>• *severe headache*<br>• *visual disturbance*<br>• *epigastric pain*<br>**OR** *SBP ≥ 160 mmHg OR diastolic blood pressure ≥ 110 mmHg*<br>**Magnesium sulfate 4 g IV with 5 g IM per buttock as initial dose (10 mg IM total), followed by 5 g IM q4 hours until delivery** |
| 12. | Is the mother bleeding?<br>□ Yes<br>□ No | *If yes, evaluate and treat per guidelines.*<br>*Prepare for a Caesarean section delivery if indicated.*<br>*Consider referral if unable to manage appropriately.* |

| | | |
|---|---|---|
| 13. | Does mother have other known disease?<br>□ None<br>□ Anemia<br>□ Chronic hypertension<br>□ Diabetes mellitus<br>□ Other condition, specify: ________________________ | *If yes, ensure appropriate treatment.* |
| 14. | Has Partograph been started?<br>□ Yes<br>□ No, will start when ≥ 4cm | *Ensure adherence to partograph guidelines.* |
| 15. | Does fetus have any concerning features/issues?<br>□ No<br>□ Fetal HR > 160 or < 100<br>□ Fetal heart rate irregular<br>□ No fetal heart beat<br>□ Malposition (breech, etc.)<br>□ Suspicion for intrauterine growth restriction<br>□ Suspected or confirmed fetal anomaly<br>□ Multiple pregnancy<br>□ Meconium stained liquor<br>□ Other condition, specify: ________________________ | *If yes, prepare for birth with additional staff assistance for delivery or call for appropriate help* |
| 16. | Does mother need referral?<br>□ Yes, Specify facility ____________________________<br>□ No | *If referral is not possible, note reason here:* |
| 17. | Does the mother have a plan for post-partum contraception?<br>□ Yes<br>□ No | *Discuss and offer family planning options to mother* |

| **3** | **JUST BEFORE AND DURING SECOND & THIRD STAGE** | |
|---|---|---|
| Date: ___________________________ | Time: ____________________________ | Collected by: _______________________ |
| Mother's Name: ___________________ | Medical record/file #:__________________________ | |

| | | |
|---|---|---|
| | **CONFIRM ESSENTIAL SUPPLIES AND SKILLS FOR MANAGEMENT OF LABOUR AND NEWBORN INCLUDING PRETERM DELIVERY** | |
| 1. | Confirm availability of essential supplies for delivery.<br>□ Sterile gloves<br>□ Soap and clean water<br>□ Delivery packs<br>□ Oxytocin<br>□ Syringes<br>□ IV fluids and giving-sets<br>□ Essential and Emergency medications | *Ensure all supplies for second and third stage are available.* |
| 2. | Assistant identified and informed to be ready to help with birth INCLUDING skilled for management of a **preterm birth**?<br>□ Yes<br>□ No | *Prepare to care for baby immediately after birth:*<br>*1. Delayed cord clamping (DCC*<br>*2. Dry baby, keep warm*<br>*3. If not breathing, stimulate and clear airway if needed*<br>*4. If still not breathing:*<br>• *clamp and cut cord*<br>• *clean airway if necessary*<br>• *ventilate with bag-and-mask*<br>*shout for help* |

| | | |
|---|---|---|
| 3. | Confirm availability of essential supplies for the newborn baby including preterm babies.<br>□ Clean towel<br>□ Suction device<br>□ Bag and mask, including appropriate size for preterm babies | |
| 4. | Does mother have delayed second stage?<br>□ Yes<br>□ No | *If Yes, prepare for appropriate interventions e.g. assisted vaginal delivery, caesarean section and appropriate neonatal care.* |
| 5. | Does the mother require Magnesium sulfate?<br>□ Yes, for pre-eclampsia<br>□ Yes, for fetal neuroprotection<br>□ No | *See indications for when to give for fetal neuroprotection and/or maternal pre-eclampsia above.*<br><br>**Magnesium sulfate 4 g IV with 5 g IM per buttock as initial dose (10 mg IM total), followed by 5 g IM q4 hours** |
| 6. | Is mother likely to deliver a preterm?<br>□ Yes<br>□ No | *Prepare for preterm birth:*<br>• *Appropriate preterm bag and mask and/or PPV are available*<br>• *Continuous warmth for unstable preterms (e.g blankets)*<br>• *Readiness to implement Kangaroo Care*<br>• *Ready to do delayed cord clamping (DCC)*<br>• *Staff assistance for delivery and resuscitation* |

| | | |
|---|---|---|
| **4.** | **SOON AFTER BIRTH - WITHIN ONE HOUR** | |
| Date: | Time: | Collected by: |
| Mother's name: | Medical record/file #: | Baby's name: |

| | | |
|---|---|---|
| 1. | Does mom have any concerning features?<br>□ High blood pressure<br>□ High temperature/signs of active infection<br>□ Heavy vaginal bleeding<br>□ Other _____________<br>□ NONE | *IF yes treat appropriately* |
| 2. | Does this baby have any concerning features?<br>□ Yes<br>□ No<br><br>If yes, mark all that apply:<br>□ Signs of respiratory distress<br>□ Signs of birth asphyxia<br>□ Fetal anomaly<br>□ Weight <2500g<br>□ Signs of Prematurity<br>□ Signs of infection<br>□ Signs of anemia<br>□ Unable to suck well<br>□ Other condition, specify: _______________________ | *If yes,*<br>• *Manage appropriately*<br>• *Call for help*<br>• *Ensure close monitoring after discharge* |
| 3. | □ Ensure baby started immediate breastfeeding and skin-to-skin contact initiated (if mother & baby are well). | For preterm encourage breast milk BUT determine appropriate mode of feeding:<br>□ Breastfeeding<br>□ Cup feeding on Expressed Breast Milk<br>□ Nasogastric Tube Feeding on Expressed Breast Milk |

| | | |
|---|---|---|
| 4. | Routine newborn care:<br>□ Delayed cord clamping<br>□ Vitamin K injection<br>□ Antibiotic eye ointment<br>□ Cord care | **Vitamin K:**<br>**Weight <1.5 kg: 0. 5 mg IM**<br>**Weight >1.5 kg: 1 mg IM** |
| 5. | Observations/anthropometric measurements taken?<br>□ Weight: ____________________ gms<br>□ Length ______________________ cm<br>□ Head circumference _____________cm<br>□ Other, specify______________ | |
| 6. | Gestational age confirmed?<br>□ Yes, _____ weeks<br>□ No<br>If yes, GA method used (weight, LMP, Ballard Score, Other):<br>________________ | *If below 37 weeks, initiate preterm care (i.e., thermal care, resuscitation as needed, weight based dosing of meds, referral, etc.) if needed.* |
| 7. | Does the baby require Antibiotics?<br>□ Yes<br>□ No | *Give if:*<br>• *antibiotics were given to mother*<br>• *baby has signs of breathing too fast (>60 breaths min) or too slow (<30 breaths min)*<br>• *chest in-drawing, grunting or convulsions*<br>• *no movement on stimulation*<br>• *baby's temperature is too cold (< 35°C and not rising after warming) or too hot (> 37.5°C)* |
| 8. | Does the baby require Antiretrovirals?<br>□ Yes<br>□ No | *Adhere to MOH guidelines on management of ARVs use in infants.* |
| | **CONFIRM NEED AND READINESS FOR CONTINUING THERMAL CARE** | |
| 9. | Confirm readiness for thermal care.<br>□ Yes<br>□ No<br>□ NOT REQUIRED | *If YES Ensure the following:*<br>• *Educate the mother on skin to skin and KMC (awareness)*<br>• *Knowledge and skills of the health provider for KMC*<br>• *Space and furniture for KMC*<br>• *Continuous warmth for unstable preterm babies, eg. Blankets* |
| 10. | Does baby require Kangaroo Mother Care?<br>□ Yes, initiated immediately or within one hour after birth<br>□ Yes, initiated more than one hour after birth<br>□ Yes, but not initiated<br>□ No | *Consider for stable preterm and low birth weight babies.* |
| 11. | Is referral of baby needed?<br>□ Yes, specify diagnosis:___________________________<br>□ No<br>If yes, specify facility referred to: __________________________ | *If yes, follow your facility's protocol for referral.* |

| **5** | **AT DISCHARGE** | |
|---|---|---|
| Date: ___________________________ | Time: __________________________ | Collected by: ______________________ |
| Mother's name: ___________________ | Medical record/file #: ___________ | Baby's name: ______________________ |
| Telephone:_______________________ | Village:_______________________________________ | |

## PRETERM BIRTH INITIATIVE - KENYA SAFE CHILDBIRTH CHECKLIST: FACILITY:_______________________

<table>
<tr><td>1.</td><td>Infant status?<br>□ Alive<br>□ Dead<br>If Alive, indicate whether:<br>□ Discharged home<br>□ Referred</td><td>If referred, specify:<br>Facility referred to:<br>______________________________<br>Condition:<br>______________________________</td><td>If discharged home, specify:<br>Date of discharge: _________________<br>Time of discharge: ________________<br>Age at discharge (days)_____________<br>Infant weight at discharge (grams)____</td></tr>
<tr><td>2.</td><td colspan="2">Is the baby feeding well?<br>□ Yes<br>□ No</td><td>If no, re-consider if needs to stay in nursery/facility for assistance in feeding<br>If okay to be discharged, give appropriate advice and recommend close follow-up</td></tr>
<tr><td>3.</td><td colspan="2">Has the baby been on antibiotics?<br>□ Yes, dose completed<br>□ Yes, dose not completed<br>□ No</td><td>Advise on completion of dose at home and recommend follow-up</td></tr>
<tr><td>4.</td><td colspan="2">Does the baby need to continue with Kangaroo Mother care?<br>□ Yes<br>□ No</td><td>If Yes:<br>• Recommend and advise on KMC at home for preterm and low birth weight babies.<br>• Recommend close follow-up including phone calls.</td></tr>
<tr><td>5.</td><td colspan="2">Length of stay(days) in the facility: _________days<br>If stay >28 days, specify reason(s):________________________</td><td></td></tr>
<tr><td>6.</td><td colspan="2">Is the mother well?<br>□ Yes<br>□ No</td><td>If No, states concerns and advise on appropriate management.</td></tr>
<tr><td>7.</td><td colspan="2">Does the mother have a plan for post-partum contraception?<br>□ Yes<br>□ No</td><td>Discuss and offer family planning options to mother</td></tr>
<tr><td>8.</td><td>□ Advise mother to report for review if the following danger signs are noted.</td><td colspan="2">Mother:<br>• Bleeding (soaking more than 2 pads per hour or otherwise concerned about the bleeding)<br>• Severe abdominal pain<br>• Severe headache or visual disturbance<br>• Breathing difficulty<br>• Fever or chills<br>• Difficulty emptying bladder<br>• Epigastric pain<br>Baby:<br>• Fast/difficult breathing<br>• Fever, unusually cold<br>• Stops feeding well<br>• Less activity than normal<br>• Whole body becomes yellow</td></tr>
<tr><td>9.</td><td colspan="2">Follow-up arranged for mother and baby?<br>□ Yes<br>□ No<br>Date of next appointment: ________________________</td><td>For preterm and/or low birth weight infants:<br>• Schedule telephone calls daily for the first 72 hours, thereafter twice weekly until baby is 28 days old.<br>• Recommend weekly follow-up in health facilities<br>• Actively follow-up will be done by phone and home visits if missed appointments</td></tr>
<tr><td>10.</td><td colspan="3">If the baby was preterm or low birth weight, request for consent for follow-up by phone calls and home visits.</td></tr>
</table>
