# Supplementary material for: Implementing the WHO Safe Childbirth Checklist modified for preterm birth: lessons learned and experiences from Kenya and Uganda
Source: BMC Health Serv Res. 2022 Mar 3;22:294. doi: 10.1186/s12913-022-07650-x (PMC8896298; doi:10.1186/s12913-022-07650-x)
Supplement: Supplementary file 3 — Additional file 3. Final mSCC Uganda Includes the final version of the modified Safe Childbirth Checklist used in the Uganda study sites [file 12913_2022_7650_MOESM3_ESM.pdf]

1 FIRST PRESENTATION OF THE MOTHER (TRIAGE)

Date: ___________________	Time: ____________________	Collected by: ___________________________________
Name: ___________________	DOB/Age: ________________	File #:____
Gravidity: _________	Parity: _________	Phone number:__________________________________

Last normal menstrual period (LNMP): __________________<br>Gestational age (GA) by LNMP: ________________________<br>GA by fundal height with tape measure, cm: ______________<br>Gestational age by ultrasound: __________________________<br>*Record all that apply.*	*If <37 weeks (preterm), admit or refer.*
Does patient have a history of preterm delivery?<br>☐ Yes<br>☐ No	*If yes, risk of preterm delivery is elevated.*
Does the mother need to be admitted for:<br>☐ High blood pressure<br>☐ High temperature/signs of active infection<br>☐ Heavy vaginal bleeding<br>☐ High or low fetal heart rate<br>☐ Preterm labor or rupture of membranes<br>☐ Active Labor<br>☐ Rupture of membranes<br>☐ Other, specify: ____________________________	*Assess for indication that mother should be admitted or referred:*<br>• *If systolic blood pressure >140 or diastolic blood pressure >90, admit or refer. Start management of pre-eclampsia.*<br>• *If ≥38°C or > 37.5°C axillary with signs of infection, admit or refer.*<br>• *If FHR>160 or <110, admit or refer. Further monitor.*<br>• *If preterm, initiate appropriate care or refer.*<br>• *If dilation more than 4 cm, admit or refer.*
Mother's status?<br>☐ Admitted<br>☐ Referred. Specify referral location: ________________<br>☐ Sent home	*If unable to manage, refer.*

 Unique identifier : ________________________

2 ON ADMISSION TO MATERNITY

Date: ____________________	Time: ____________________	Collected by: __________________________________	
Name: ____________________	DOB/Age: ________________	File#:____________________	Phone #:________________

FOR MOTHERS <34 WEEKS (IF NOT, SKIP TO NEXT SECTION)

Does mother need antenatal corticosteroids?<br>☐ Yes, given<br>☐ No	*Give antenatal corticosteroids per national protocol (if no evidence of maternal infection and delivery is imminent within 24 hours to seven days).*
Does mother need tocolytics (medication to stop uterine contractions)?<br>☐ Yes, given<br>☐ No	*Consider tocolytics (e.g. nifepidene) if giving steroids.*
Does mother need antibiotics?<br>☐ Yes, given<br>☐ No	*Give antibiotics:*<br>• *PPROM 28-34 weeks*<br>• *temperature > 38°C or > 37.5°C axillary*<br>• *foul-smelling vaginal discharge*
Does mother need magnesium sulfate for fetal neuroprotection?<br>☐ Yes, given<br>☐ No	*Consider magnesium sulfate for fetal neuroprotection if imminent preterm birth delivery between 28-32 weeks of gestation.*

ALL MOTHERS (all gestational age)

Does mother need antibiotics?<br>☐ Yes, given<br>☐ Yes, already given for preterm indication<br>☐ No	*Give antibiotics if any of the following:*<br>• *temperature > 38°C or > 37.5°C axillary*<br>• *foul-smelling vaginal discharge*<br>• *rupture of membranes > 12 hours*
Does mother need anti-malarials?<br>☐ Yes, given<br>☐ No	*Give anti-malarials if mother has temp >38°C or > 37.5°C axillary and:*<br>• *positive malaria smear*<br>• *meets other diagnostic criteria for malaria*
Does mother need antiretroviral medicine?<br>☐ Yes, given<br>☐ No	*Give if mother is HIV+ and in labor.*<br>*If on ARVs, remind mother to take her medicine.*
Does mother need magnesium sulfate for pre-eclampsia?<br>☐ Yes, given<br>☐ Yes, already given for fetal neuroprotection<br>☐ No	*Give if any of:*<br>• *diastolic blood pressure ≥ 110 mmHg and 3+ proteinuria*<br>• *diastolic blood pressure ≥ 90 mmHg, 2+ proteinuria AND if any: severe headache, visual disturbance, or epigastric pain*
Does mother need Anti- hypertensive treatment?<br>☐ Yes, given<br>☐ No	*Give if systolic blood pressure ≥ 160 mmHg.*
Does mother have significant bleeding?<br>☐ Yes<br>☐ No	*If yes, follow clinical guidelines for management.*<br>*Prepare for obstetric hemorrhage and neonatal compromise.*<br>*Consider referral if unable to manage appropriately.*

Does mother have a known significant disease? *(tick all that apply)*<br>□ None known<br>□ Severe anemia<br>□ Chronic hypertension<br>□ Diabetes mellitus<br>□ Other condition, specify: : _________________________	*If yes, ensure appropriate treatment.*
Does fetus have any concerning features? *(tick all that apply)*<br>□ No<br>□ Stillbirth<br>□ Malposition (breech, etc.)<br>□ Fetal HR > 160 or <110<br>□ Multiple pregnancy<br>□ Meconium stained fluid<br>□ Suspicion for intrauterine growth restriction<br>□ Fetal anomaly<br>□ Other condition, specify: _______________________	*If yes, prepare for birth with additional staff assistance for delivery.*
Does mother need referral?<br>□ Yes, organized and contacted<br>□ No<br>Reason for referral:_________________<br>Referral location: __________________<br>If referral is not possible, note reason here: ______________	
□ Confirm supplies available to clean hands and wear gloves for each vaginal exam.	
□ Birth companion encouraged to be present at birth.	
□ Counsel mother or companion to call for help if needed.	*Call for help if:*<br>• *Bleeding*<br>• *severe abdominal pain*<br>• *severe headache*<br>• *visual disturbance*<br>• *urge to push or difficulty emptying bladder*
□ Confirm that mother has a plan for post-partum contraception. Does she want a Long Acting reversible Contraceptive (LARC) method or sterilization immediately post-partum?	*If yes, determine if an implant or intrauterine device (IUD) is available or confirm plan for sterilization. Prepare for immediate post-partum insertion of the appropriate method or postpartum sterilization.*

3 JUST BEFORE PUSHING OR BEFORE CAESAREAN

Date: ____________________	Time: ____________________	Collected by: ____

□ Assistant identified and informed to be ready to help at birth if needed.	*Assistance needed if any of the following:*<br>• *Preterm birth*<br>• *FHR > 160 or < 110*<br>• *Meconium present*<br>• *Other anticipated problem for mother or infant*
Does mother need antibiotics?<br>□ Yes, given<br>□ No	*Give antibiotics:*<br>• *PPROM 28-34 weeks*<br>• *rupture of membranes after 34weeks > 12 hours*<br>• *Temperature > 38°C or > 37.5°C axillary*<br>• *Foul-smelling vaginal discharge*<br>• *Planned C-section*
Does mother need magnesium sulfate?<br>□ Yes, for pre-eclampsia, given<br>□ Yes, for fetal neuroprotection, given<br>□ No	*Give if:*<br>• *diastolic blood pressure ≥ 110 mmHg and 3+ proteinuria*<br>• *diastolic blood pressure ≥ 90 mmHg, 2+ proteinuria AND if any: severe headache, visual disturbance, or epigastric pain*<br>*OR for fetal neuroprotection if imminent delivery 28-32 weeks*
Does mother need anti-hypertensive treatment?<br>□ Yes, given<br>□ No	*Give if systolic blood pressure ≥ 160 mmHg*
□ Confirm essential supplies at bedside for mother<br>*Tick box if all are available. Circle any of the following that are missing:*<br>• Gloves<br>• Soap and clean water or alcohol-based handrub<br>• Oxytocin 10 units in a syringe<br>• Other uterotonic, if oxytocin not available	*Prepare to care for mother immediately after birth:*<br>1. *Exclude 2nd baby*<br>2. *Give oxytocin within 1 minute, or other uterotonic if oxytocin not available*<br>3. *Deliver placenta safely according to AMTSL*<br>4. *Check uterine tone after placenta is delivered*<br>5. *Confirm uterus is contracted*
□ Confirm essential supplies at bedside for baby<br>*Tick box if all are available. Circle any of the following that are missing:*<br>• Clean towel<br>• Sterile blade to cut cord<br>• Suction device<br>• Bag and mask	*Prepare to care for baby immediately after birth:*<br>1. *Dry and simulate baby and keep warm*<br>2. *If not breathing: stimulate and clear airway*<br>3. *If still not breathing:*<br>- *cut cord*<br>- *begin positive pressure ventilation with bag and mask*<br>- *shout for help*
□ Confirm ready to initiate Kangaroo Care if preterm	

4 SOON AFTER BIRTH - WITHIN ONE HOUR

Date: ____________________	Time: ____________________	Collected by: ____

Delivery mode: ☐ Vaginal delivery<br>☐ Operative vaginal delivery<br>☐ Caesarean	Labor mode: ☐ Induced<br>☐ Spontaneous

Multiple gestation? ☐ Yes<br>☐ No<br>*If yes, specify information for each baby.*	Infant date/time of birth:<br>____________________	Infant weight at birth:<br>____________________	Infant gender: ☐ Male<br>☐ Female

☐ Alive<br>☐ Fresh stillbirth<br>☐ Macerated stillbirth<br>☐ Early neonatal death	*If infant dies, offer emotional support services to mother.*
Is mother bleeding too much?<br>☐ Yes, shout for help<br>☐ No	*If bleeding abnormally:*<br>• *Massage uterus after placenta is delivered*<br>• *Consider additional uterotonic*<br>• *Start intravenous line*<br>• *Look for lacerations, examine placenta, and keep mother warm*<br>• *Treat cause*<br>• *Take blood for grouping and cross match*
Does baby have any concerning features? *(check all that apply)*<br>☐ No<br>☐ Fetal anomaly<br>☐ Size <2500g<br>☐ Signs of infection<br>☐ Signs of respiratory distress<br>☐ Signs of anemia<br>☐ Signs of birth asphyxia<br>☐ Other condition, specify:	*If yes, shout for help. Once stabilized, consider transfer to newborn unit or higher level care.*
Does mother need antibiotics?<br>☐ Yes, given<br>☐ No	*Give antibiotics if any of the following:*<br>• *Placental manually removed*<br>• *Mother's temperature > 38°C or > 37.5°C axillary AND chills or foul-smelling vaginal discharge*<br>• *3rd degree tear*<br>• *Assisted delivery*<br>• *C-section*<br>• *PROM<12 hours/PPROM 28-34weeks*
Does mother need magnesium sulfate for pre-eclampsia?<br>☐ Yes, given<br>☐ No	*Give if any of:*<br>• *diastolic blood pressure ≥ 110 mmHg and 3+ proteinuria*<br>• *diastolic blood pressure ≥ 90 mmHg, 2+ proteinuria AND if any: severe headache, visual disturbance, or epigastric pain*
Does mother need anti-hypertensive treatment?<br>☐ Yes, given<br>☐ No	*Give if systolic blood pressure ≥ 160 mmHg*
Does baby need referral?<br>☐ Yes<br>☐ No<br>Reason for referral:________________________________<br>Referral location: ________________________________<br>If referral is not possible, note reason here: ______________	*If yes, follow your facility's protocol for referral.*

<table>
<tr>
<td>Gestational age:
☐ GA by LNMP ________
☐ Weight __________gms

If indicated:
GA by Ballard _____________</td>
<td colspan="2">If below 37 weeks, initiate preterm care (i.e., resuscitation with PPV, Kangaroo Care, referral) if needed.

If baby <2500 gms OR <37 weeks, do Ballard exam to confirm gestational age</td>
</tr>
<tr>
<td>Does baby need: (tick all that apply)
☐ Special care and monitoring
☐ Antiretrovirals
☐ Antibiotics</td>
<td>Arrange special care and monitoring if:
• born more than 1 month early
• has birth weight <2500 grams
• needs antibiotics
• required resuscitation
• baby has danger sign

Give antiretrovirals if mother is HIV+.</td>
<td>Give antibiotics if:
• antibiotics were given to mother
• baby has signs of breathing too fast (>60 breaths min) or too slow (<30 breaths min)
• Chest in-drawing, grunting or convulsions
• No movement on stimulation
• Baby's temperature is too cold (< 35°C and not rising after warming) or too hot (> 38°C)</td>
</tr>
<tr>
<td>Breastfeeding and skin-to-skin initiated?
☐ Yes, immediately or within one hour after birth
☐ Yes, more than one hour after birth
☐ No</td>
<td colspan="2">Consider supplemental feeding if baby unable to breastfeed.</td>
</tr>
<tr>
<td>Does baby need Kangaroo care?
☐ Yes, initiated immediately or within one hour after birth
☐ Yes, initiated more than one hour after birth
☐ No</td>
<td colspan="2">Consider for preterm or low birth weight baby.</td>
</tr>
<tr>
<td colspan="3"></td>
</tr>
<tr>
<td>☐ Counsel mother or companion to call for help if danger signs are present.</td>
<td>Mother has any of the following:
• bleeding
• severe abdominal pain
• severe headache
• visual disturbance
• breathing difficulty
• fever chills
• difficulty emptying bladder
• convulsions</td>
<td>Baby has any of the following:
• fast or difficulty breathing
• fever
• unusually cold
• stops feeding well
• less activity than normal
• whole body becomes yellow
• convulsions</td>
</tr>
<tr>
<td>Does mother want Long Acting reversible Contraceptive (LARC) method or sterilization immediately post-partum?
☐ Yes
☐ No</td>
<td colspan="2">Confirm that mother has a plan for post-partum contraception. If mother wants LARC, determine if an implant or intrauterine device (IUD) is available. Prepare for immediate post-partum insertion of the appropriate method.
If wants sterilization, confirm plan and availability of this service.</td>
</tr>
</table>

5 BEFORE DISCHARGE

Date: ____________________	Time: __________________	Collected by: ______________________________________

Infant disposition/status at discharge?<br>□ Alive, discharged home<br>□ Stillbirth<br>□ Early neonatal death<br>□ Referred to another facility : _____________________	*If infant dies, offer emotional support services to mother.*<br><br>*If multiple gestation, specify for each baby.*
Does mother have concerning features?<br>□ Bleeding too much<br>□ Needs antibiotics<br>□ Elevated blood pressure (>140/90)<br>□ Other: __________________________________<br>□ None	*If pulse >110 beats/minute and systolic blood pressure <90mmHg.*<br>• *Start IV*<br>• *Keep mother warm*<br>• *Consider blood transfusion*<br>• *Treat cause*<br><br>*Give antibiotics to mother if any of the following:*<br>• *Mother's temperature > 38°C or > 37.5°C axillary*<br>• *Foul-smelling vaginal discharge*<br>• *Chills*<br><br>*IF YES to any, treat and delay discharge*
Does baby have concerning features?<br>□ Needs antibiotics<br>□ Not feeding well<br>□ Needs Kangaroo Care<br>□ None	*Give antibiotics to baby if any of the following:*<br>• *baby has signs of breathing too fast (>60 breaths/min) or too slow (<30 breaths/min)*<br>• *Chest in-drawing, grunting or convulsions*<br>• *No movement on stimulation*<br>• *Baby's temperature is too cold (< 35°C and not rising after warming) or too hot (> 38°C)*<br>• *Stopped breastfeeding well*<br>• *Umbilical redness extending to skin or draining pus*<br><br>*If baby feeding well, emphasize exclusive breastfeeding. If not, reconsider discharge plan and consider supplemental feeding.*<br><br>*Consider Kangaroo Care for preterm or low birth weight baby and recommend close follow up or delay discharge*
Family planning options discussed and offered to mother?<br>□ Yes, implant given<br>□ Yes, IUD given<br>□ Yes, Depo given<br>□ Yes, pills given<br>□ Yes, sterilization done<br>□ Not discussed<br>□ Discussed but not offered prior to discharge<br>□ Discussed and offered, but mother declined	• *If implant or IUD desired, place now and make follow- up plan.*<br>• *If Depo desired, give injection now and discuss follow-up schedule.*<br>• *If pills desired, give now and discuss follow-up plan.*
□ Arrange follow-up for mother and baby and counsel mother and companion to call for help after discharge if danger signs appear after discharge:	*Mother has any of the following danger signs:*<br>• *Bleeding*<br>• *severe abdominal pain*<br>• *severe headache*<br>• *visual disturbance*<br>• *breathing difficulty*<br>• *fever chills*<br>• *difficulty emptying bladder*<br>• *convulsions*<br><br>*Baby has any of the following danger signs:*<br>• *fast or difficulty breathing*<br>• *fever*<br>• *unusually cold*<br>• *stops feeding well*<br>• *less activity than normal*<br>• *whole body becomes yellow*<br>• *convulsions*
