# Supplementary figures and images for: Implementing the WHO Safe Childbirth Checklist modified for preterm birth: lessons learned and experiences from Kenya and Uganda
Source: BMC Health Serv Res. 2022 Mar 3;22:294. doi: 10.1186/s12913-022-07650-x (PMC8896298; doi:10.1186/s12913-022-07650-x)

Additional File 4: Study timeline and roll-out of intervention components

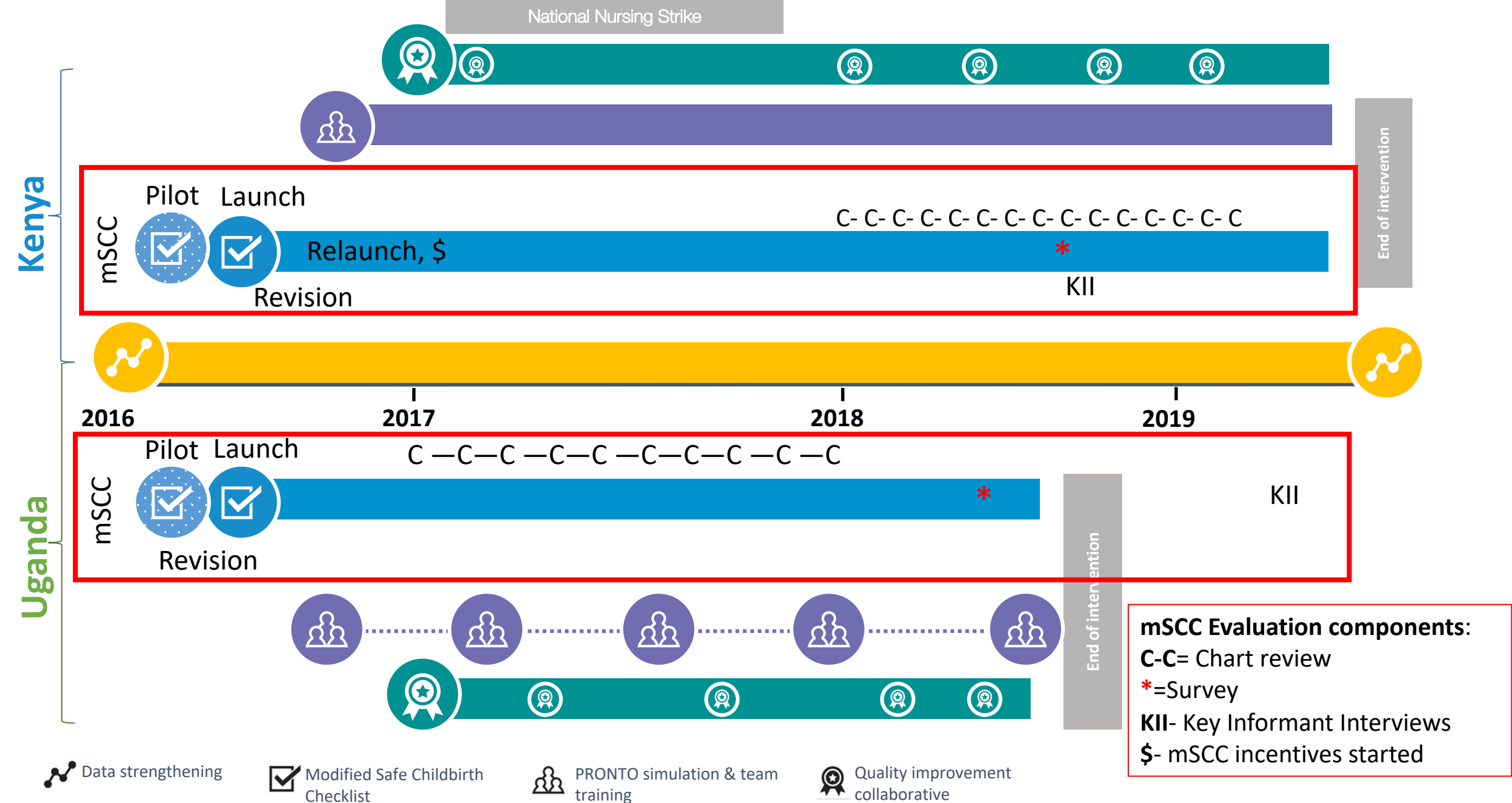

Supplement: Supplementary file 4 — Additional file 4. Study Timeline Includes a timeline which shows implementation of all interventions as well as data collection activities for this analysis [file 12913_2022_7650_MOESM4_ESM.pdf]
