# Supplementary material for: Implementing the WHO Safe Childbirth Checklist modified for preterm birth: lessons learned and experiences from Kenya and Uganda
Source: BMC Health Serv Res. 2022 Mar 3;22:294. doi: 10.1186/s12913-022-07650-x (PMC8896298; doi:10.1186/s12913-022-07650-x)
Supplement: Supplementary file 5 — Additional file 5. HW mSCC Feedback Survey Tool Includes the survey developed and administered in both countries to elicit feedback about the modified Safe Childbirth Checklist [file 12913_2022_7650_MOESM5_ESM.docx]

**SURVEY QUESTIONNAIRE (HEALTHWORKERS)**

mSCC Programmatic Feedback Survey

User Feedback

*Thank you for taking a few minutes to answer questions about the Safe Childbirth Checklist! We want this tool to assist you in your work. If there are things that you like, that you do not like, change that you would like to see please let us know. We are looking for honest, constructive feedback so please by candid and complete. Responses are completely anonymous and will help us to make changes for the future.*

**Demographics**

**Select your profession:**

Midwife

Nurse

CO

MO

Obstetrciain Gynecologist

Pediatrician

Nursing Student

Nursing Assistant

**Age:**

18 – 80

**Select your sex:**

Male

Female

**How many years have you practiced in your profession?**

0 – 40

**Length of time at your current/most recent facility.**

<1 to 30+

**Have you used the Safe Childbirth Checklist?**

Yes | No

**If no, have you heard of the Safe Childbirth Checklist?**

Yes | No

**If yes, when have you used it? (check all that apply)**

When women present at the facility

When women are admitted to the maternity ward

During second and third stages of labor

In the immediate post-partum period

At discharge

**Where do you get the checklist from?**

Nursing desk

Inpatient chart

Front desk

**Do you ever run out of checklists?**

**If yes, do you know who to go to get more?**

**Are they printed double-sided or single-sided?**

**Is it important to include the checklist in the patient chart?**

**An electronic checklist would be:**

More helpful than the paper version

The same

Less helpful than the paper version

**If the checklist was electronic, would you prefer it on:**

A tablet

Your own phone

A laptop

Desktop computer

**Did you know that the checklist has clinical prompts**

Yes/No

**Do you think the clinical prompts are:**

Very helpful

Somewhat helpful

Not helpful

Unhelpful

**Do the prompts need improvement?**

Open ended.

*Please rate the following statements on a scale from strongly agree to strongly disagree.*

**The checklist is easy to read**

Strongly Agree

Agree

Disagree

Strongly Disagree

N/A

**The checklist is easy to follow**

Strongly Agree

Agree

Disagree

Strongly Disagree

N/A

**The checklist repeats information written in the inpatient chart**

Strongly Agree

Agree

Disagree

Strongly Disagree

N/A

**The checklist repeats information written in the maternity register**

Strongly Agree

Agree

Disagree

Strongly Disagree

N/A

**The checklist makes my job easier**

Strongly Agree

Agree

Disagree

Strongly Disagree

N/A

**The checklist has helped you in clinical decision making in at least once instance**

Strongly Agree

Agree

Disagree

Strongly Disagree

N/A

**Of the five pause points, which do you find most useful?**

First presentation of the mother (triage)

On admission to maternity

Just before pushing or before cesarean

Soon after birth – within one hour

Before discharge

**When using the checklist, do you:**

Read the checklist and then do an action

Do an action and then check it off

A bit of both

**The safe childbirth checklist has helped me to identify cases of….**

**Preterm labor**

Strongly Agree

Agree

Disagree

Strongly Disagree

N/A

**Preeclampsia**

Strongly Agree

Agree

Disagree

Strongly Disagree

N/A

**Multiple Gestation**

Strongly Agree

Agree

Disagree

Strongly Disagree

N/A

**Maternal Infection**

Strongly Agree

Agree

Disagree

Strongly Disagree

N/A

**I have referred more cases to different facilities since using the SCC**

Strongly Agree

Agree

Disagree

Strongly Disagree

N/A

**I have identified babies as preterm as a result**

Strongly Agree

Agree

Disagree

Strongly Disagree

N/A

**I prepare my work station for a birth every time because mSCC**

Strongly Agree

Agree

Disagree

Strongly Disagree

N/A

**I think the checklist is useful for:**

Normal births

Complicated births

Teaching students

Working by myself

**The most helpful format for the checklist to be presented in would be….**

Something on the wall

My phone

Tablet

Talks back to me

Laminated copies

White board

something with signal lights (green/red)

Paper

If this were available on a tablet, would this be easier?

If you completed the checklist on a tablet, would it be important to be able to print it when complete?

**I feel more confident managing cases…**

Strongly Agree

Agree

Disagree

Strongly Disagree

N/A

**I use the checklist for every birth.**

Strongly Agree

Agree

Disagree

Strongly Disagree

N/A

**If I use the checklist multiple times, I know what I do and I don’t need to use it anymore.**

Strongly Agree

Agree

Disagree

Strongly Disagree

N/A

**If there were no financial incentives, would you still use this tool?**

Yes

No

Not sure

**My facility…**

Always has the supplies I need

Sometimes has the supplies I need

Never has the supplies I need

**I learned something I did not know before by reading the checklist.**

Strongly Agree

Agree

Disagree

Strongly Disagree

N/A

**I changed my clinical decision after using the checklist.**

Strongly Agree

Agree

Disagree

Strongly Disagree

N/A

**The checklist helped me in a situation where I did not know what to do with my patient.**

Strongly Agree

Agree

Disagree

Strongly Disagree

N/A

Open Ended

**In your own words, what do you think about the Safe Childbirth Checklist?**

**In your own words, how could the Safe Childbirth Checklist be improved?**

**What is the one thing that would make your job easier?**

**What do you like most about your job?**

**What do you like least?**

**If you could change one thing about supply availability, what would it be?**

**If you were pregnant, would you give birth at this facility?**

Yes

No

Only if there were no other option
