# Supplementary material for: Implementing the WHO Safe Childbirth Checklist modified for preterm birth: lessons learned and experiences from Kenya and Uganda
Source: BMC Health Serv Res. 2022 Mar 3;22:294. doi: 10.1186/s12913-022-07650-x (PMC8896298; doi:10.1186/s12913-022-07650-x)
Supplement: Supplementary file 6 — Additional file 6. Kenya HW Interview Guides (English) Ver 1.0 March 2018 Includes the interview guide used in Kenya for healthworker interviews [file 12913_2022_7650_MOESM6_ESM.pdf]

PTBI - K PROJECT: KEMRI/UCSF/MIGORI COUNTY
APPENDIX 7: PROCESS EVALUATION INTERVIEW GUIDES

INTERVIEW GUIDE – HEALTH WORKERS	
Ice-breaking, Warm up & Messaging<br>▪ *General Newborn Care*<br>▪ *Care for Pre-terms*	Ask & NOTE **respondent biodata**:<br>- Respondent Name, Age, Gender, Religion, Ethnicity<br>- Facility Name, Position/ role, Length or duration of work<br>How would you describe the state of newborn health at your facility? In this region?<br>Can you please take me through what is normally done for high-risk mothers and babies? *Probe for pre-during-after childbirth, reference to HRB guidelines,etc*<br>What kind of care is given for pre-term babies? Probe for both hospital care & home care<br>Who do you work with mostly in newborn care? *Probe internal & external support systems + what exactly*
Knowledge and management of preterms and newborns	What is your experience with pre-term babies? *Probe for confidence levels of PTB management*<br>How does your facility deal with pre-term babies?<br>What do you know about the management of pre-term babies? *Probe for knowledge sources – who trained, how useful & how comprehensive – all NC elements)*<br>How do you determine that a pre-term baby is ready for discharge?<br>What usually happens after discharge of a pre-term baby? *Probe for hospital systems + any follow-up mechanisms*<br>*What do you think explains the high death rates of pre-terms after discharge?*<br>*What do you think can be done to reduce this?*
Service User (community) KAP	What does the community know and think about pre-term babies?<br>How do you /they handle them while at the hospital?<br>What happens when they leave the hospital *(Probe for issues around care after discharge, ask for case studies, any follow up cases,etc)*
PTBi Intervention Components<br>▪ *Training & Mentorship*	What do you know about PTBi? How did you get to know about it? *Probe project knowledge + how it was introduced to them*<br>Can you help me draw a scenario of pre-term baby care before and after the PTBi project started?<br>How has PTBi worked with you and/or your facility on care for pre-term babies?<br>What do you think of the project? *Probe for the good, bad & ugly + why*<br>How confident do you feel about managing pre-term babies after PTBi?<br><br>What do you know about PTBi mentorship?<br>How does it happen? Probe for modalities, frequency

	What exactly do the mentors do? And healthworkers?<br>What are your own experiences with regard to training & mentorship?<br>What do you think about the usefulness of this approach? *Probe for reservations if any & why*<br>Can you give me some examples of when you think mentorship was useful? Why<br>What is working well in the training & mentorship process that you think should continue & be strengthened?<br>In your opinion, what is not working well that could be improved?
PRONTO	What do you know about PRONTO?<br>How exactly does it happen?<br>How is this training similar or different from other trainings? How does PRONTO training fit within our context?<br>What knowledge and/or skills have you or your colleagues learned from PRONTO? *Ask for example/case study of how they've used PRONTO knowledge/skills.*<br>Tell me what you remember about Module 1 and 2 trainings.<br>How do you compare them with the PRONTO mentoring visits? How is each one of them useful? Which do you prefer & why?<br>What worked for you? Highlights & experiences from PRONTO?<br>In your opinion, what is not working well that could be improved?
Modified Safe Childbirth Checklist (mSCC)	What do you know about the mSCC? *Probe for how it was introduced & how staff felt about it*<br>How do you [or your colleagues] use the checklist? *Probe for effect on work/practice, issues of compliance,etc*<br>What are your [honest] views about it and its use? *Probe for what's liked & disliked about it, perceived usefulness*<br>What support systems [if any] exist to ensure mSCC use? *Probe for whether they feel supported, by who, how,etc*<br>How can the checklist be made more useful and easy to implement?<br>Is there anything else you'd like to say about the checklist?
Quality Improvement (QI)	What do you know about QI?<br>What exactly happens during QI sessions?<br>What are your experiences in the learning sessions? What about at the facilities?<br>– probe for highlights especially & perceived usefulness<br>I am aware of the different QI activities/ projects, including PTBi – how do you deal with this? *Probe coping strategies, effects on work,etc?*<br>What have been the implementation challenges of QI?<br>Apart from QI how do you generally use the different interventions – do they complement each other or not?

Data Strengthening (DS)	How does data strengthening relate to your day-to-day work?<br>What do you know about project's DS process? How exactly did it happen, what was done, when and what similarities or differences did you note? Who did what? *Probe input from study team & providers or other staff*<br>Please tell me what you know about prematurity. What about PTB related variables – how has this changed?<br>How do healthworkers use data from QI and elsewhere? What do they use it for?<br>What do you think about the facility reports? How are they being used & what else would you like to see?<br>What are your experiences with data strengthening – probe implementation successes & challenges<br>Has there been any success with integration to district and national data systems?
Impact<br>*Sustainability*<br>*Spillovers /Unintended Consequences*	What do you think are PTBi's project success? What explains this success? *Probe for areas of consolidation*<br>What about the project challenges overall? What challenges came up generally and how were they addressed?<br>What do you think are the project's weaknesses – what should be improved?<br>What are your thoughts in regarding to the continuity of these interventions? *Probe for sustainability ideas*<br>*I*s there anything – either good or bad – that was not intended but has happened as a result of PTBi? *Probe what, how, why*<br>In your opinion what can we learn from the PTBi experience?
Wrap up	• As we conclude, is there anything else you'd like to tell us in regards to what we've just discussed? Anything that you think would be useful for this evaluation or for preterm/newborn care?<br>**THANK YOU**

INTERVIEW GUIDE – HOSPITAL MANAGER - clinical	
Intros & Background	Bio data:<br>- Name, Gender, Qualifications, Position/ role<br>- Facility Name, Length or duration of work
Knowledge and management of preterm and newborns	What is the experience of this hospital with pre-term babies? *Probe for numbers & related outcomes*<br>As a hospital, how prepared and equipped do you feel in handling pre-term babies?<br>How is care for newborns organised at this facility? How do your staff deal with pre-term babies? *Probe for issues around ALL health system blocks – medicines, vaccines & equipment, staffing, financing, HMIS data systems,etc*<br>What systems and measure are in place here to ensure the survival of newborns in general & preterms in particular? *Probe neonatal units/corners & functionality, HW skills, issues around skilled HW rotation on wards,etc*<br><br>A lot has been said about high mortality among PTB after discharge, what is the experience of this hospital?<br>What do you think explains the high death rates of pre-terms after discharge?<br>What do you think can be done to reduce this?<br>How do you [or your staff] determine that a pre-term baby is ready for discharge?<br>What usually happens before and after discharge of a pre-term baby? *Probe for hospital systems + any follow-up mechanisms*
Service User (community) KAP	What does the community know and think about pre-term babies?<br>How do you they handle them while at the hospital?<br>What happens when they leave the hospital *(Probe for issues around care after discharge, ask for case studies, any follow up cases,etc)*
PTBi	What do you know about the project called PTBi? *Probe project knowledge, source + how it was introduced, level of consultation,etc*<br>How has PTBi worked with you and/or your facility?<br>Generally what do you think of the project? *Probe for the good, bad & ugly + why*
PRONTO	**PTBi has 4 main components in its intervention, and I'd like to hear your views and/or experience on each**<br>What do you know about PRONTO training for providers? *Explain each component if manager doesn't know & then ask*<br>What do you think of the method of training, especially within our context?<br>How has it helped your staff?<br>What do you think of its usefulness? Feasibility? Continuity?

PTBI - K PROJECT: KEMRI/UCSF/MIGORI COUNTY
APPENDIX 7: PROCESS EVALUATION INTERVIEW GUIDES

Modified Safe Childbirth Checklist (mSCC)	What do you know about the mSCC?<br>How has it been operationalized at your facility?<br>What are your [honest] views about it and its use? *Probe for what's liked & disliked about it, perceived usefulness*<br>What support systems [if any] exist to ensure mSCC use? *Probe for whether hospital feels supported, by who, how,etc*<br>How can the checklist be made more useful and easy to implement?<br>Is there anything else you'd like to say about the checklist?
Quality Improvement (QI) Training & Mentorship	What do you know about QI training & mentorships?<br>What are the experiences of you and/or your staff with QI? in the learning sessions?<br>I am aware of the different QI activities/ projects, including PTBi – how do you deal with this? *Probe coping strategies, effects on work,etc?*<br>What have been the implementation challenges of QI/ mentorship & training<br>Apart from QI how do you generally use the different interventions – how do they complement each other?
Data Strengthening (DS)	What systems or structures does this hospital have in place for data? How do you they work?<br>How do your staff use data systems (e.g. for QI) in their daily practice? What do they use it for?<br>What about management – does this data feed into management & policy decisions? If yes, how? If no, why?<br>What do you think about the facility reports? How are they being used & what else would you like to see?<br>How does this relate now to PTBi's data strengthening part?<br>What are your experiences with PTBi's data strengthening – probe implementation successes & challenges<br>Has there been any success with integration to County/sub county and national data systems? What do you think of it's continuity?<br>What is working? What would you like to see improved?
Perceived Impact	Which of the four components (PRONTO, QI, mSCC & Data) is likely to continue/be mainstreamed & why?<br>What effect has the project had on this hospital? – *Probe both positive & negative e.g. new skills or heavier workloads respectively*<br>What has worked well? What would you like to see improved?
Concluding Remarks	• As we conclude, is there anything else you'd like to tell us in regards to what we've just discussed? Anything that you think would be useful for this evaluation or for preterm/newborn care?<br>**THANK YOU**
