# Supplementary material for: Implementing the WHO Safe Childbirth Checklist modified for preterm birth: lessons learned and experiences from Kenya and Uganda
Source: BMC Health Serv Res. 2022 Mar 3;22:294. doi: 10.1186/s12913-022-07650-x (PMC8896298; doi:10.1186/s12913-022-07650-x)
Supplement: Supplementary file 7 — Additional file 7. Uganda_IDI guide for post-implementation mSCC and Qi data collection Includes the interview guide used in Uganda for healthworker interviews [file 12913_2022_7650_MOESM7_ESM.docx]

**Strengthening selected interventions to improve birth outcomes and reduce morbidity and mortality of preterm infants in health facilities in Migori County, Kenya and Busoga Region, Uganda: An implementation science study**

**In-depth interview guide for process evaluation of the implementation of modified Safe Childbirth Checklist and Quality Improvement study components**

| **Participant particulars:**  Name of hospital………………………………; Type of hospital…………………………………..  Age of respondent……………..  Gender………………………….  Cadre……………………………  Years of service in that hospital……………… |
| --- |
| 1. What do you know about the modified Safe Childbirth Checklist (mSCC)?    - *Probe for how staff felt about it* 2. What is your comment on how (process) the mSCC was introduced in the health facility? 3. What are your/colleagues’ experiences in using the checklist?    - *Probe for effect on work/practice and completeness,*    - *Probe for what’s liked & disliked about it, perceived usefulness per pause point* 4. What are some of the challenges you or your colleagues faced while using the checklist? 5. How can the checklist be made more useful and easy to implement (user friendly)?    - *Probe for possible modifications (as aid vs. incorporating in the patient chart; and modifications per pause point )* 6. How can the mSCC be sustained in health facilities?   *Probe if there is any other checklist that is routinely used, and what has helped its sustenance?(If no checklist is mentioned, then ask about the partograph)*   1. Is there anything else you’d like to say about the mSCC checklist? |
| 1. What do you know about QI activities under PTBI? 2. What exactly happened during QI meetings/sessions at the facilities?    - *Probe for perceived usefulness* 3. What are your experiences in the learning sessions?    - *Probe for perceived usefulness* 4. What do you think has been the role of hospital leadership in implementing the QI approach?    - *Probe for any support from hospital leadership* 5. What have been the implementation challenges/barriers of QI? 6. What do you think can be done in future to overcome such challenges in your facility? 7. Is there anything else you would like to say about QI? |
