# Supplementary material for: Implementing the WHO Safe Childbirth Checklist modified for preterm birth: lessons learned and experiences from Kenya and Uganda
Source: BMC Health Serv Res. 2022 Mar 3;22:294. doi: 10.1186/s12913-022-07650-x (PMC8896298; doi:10.1186/s12913-022-07650-x)
Supplement: Supplementary file 9 — Additional file 9. TIDieR Checklist mSCC Includes the completed TIDieR checklist for the implementation of the mSCC described in this manuscript [file 12913_2022_7650_MOESM9_ESM.pdf]

TIDieR

PTBi EA modified Safe Childbirth Checklist

**Details:**	All sites in the PTBi EA intrapartum package trial received data strengthening and implementation of the modified Safe Childbirth Checklist (mSCC). The mSCC was modified to include a pre-admission triage pause point, include additional elements for the identification and management of preterm labor and preterm birth, and aligned with Kenyan and Ugandan national obstetric and neonatal guidelines.
**Why:**	The checklist was provided to all sites as a reminder of the essential evidence-based practices that should be practiced for all births. While these practices are known, they are often not practiced, and a checklist offers a simple low-cost way to reminder providers of them.
**What (material):**	All sites received printed paper-based checklists which were included in patient charts. Checklist versions for each country are included as Additional Files attached to the article. Training consisted of a line-by-line review of each checklist item, to ensure understanding and answer any questions about the practices.
**What (procedures):**	Checklists were printed for each facility. Per feedback from providers these were integrated directly into patient medical charts. Initial training was provided by the study team, subsequent training provided on-the job- by facility staff to peers. Providers ticked each item on the paper checklist when they completed it.
**Who provided:**	Checklist completion was done by the frontline provider attending to women at each point of care - at maternity ward arrival/triage, at admission, during labor, after delivery, and before discharge. Different section may be completed by different providers. All providers were expected to complete the checklist whether they attended a group training or had a brief verbal orientation by a peer.
**How (mode of delivery; individual or group):**	Checklists were completed individually for each delivering mother. Multiple providers might collaborate in completing the checklist over the course of care.
**Where:**	The checklist was implemented in public and private not-for-profit facilities in Kenya and Uganda in maternity and postnatal wards. Checklists were added to existing patient medical charts; in instances when these were out-of-stock, checklists may have been used alone. The PTBi-EA did printing and distribution of checklists, which were in English as are medical charts in each country. Checklist completion depended on their availability and the availability of writing implements. Appropriate filing required medical charts.
**When and how much:**	The study team provided an initial training and refreshers twice annually. Outside these, orientation was on the job by peers for new staff.<br>One checklist was completed per delivering mother and the checklist was part of standard care for all births.
**Tailoring:**	No individual tailoring was done per se, however, Kenya and Uganda implemented slightly differently according to their needs and each had its own version of the checklist aligned with national standards and agreed on by local stakeholders. In addition, in Kenya, the entire checklist was placed at the front of the medical chart and providers were paid a small incentive (USD 0.50) for completion. In Uganda, the pause points were distributed in the chart to align with other care documentation.<br>The checklist was implemented in all sites in the larger cRCT - control sites received only the checklist and data strengthening activities. Intervention sites also received PRONTO simulation and team training, clinical mentoring and participated in QI collaboratives, all of which reinforced checklist use.

PTB-EA modified Safe Childbirth Checklist

Modification: The mSCC was not intended as a stand-alone tool, but in some instances facilities ran out of patient charts and used just an mSCC in its place.

How well (planned): Adherence to checklist use was assessed by completion monitoring. Since Kenya paid a small incentive, they assessed all charts for checklist completion and had consistently high rates high rates - over 70% for all pause points. In Uganda a 10% sample of all charts was assessed for checklist completeness and overall completion rates ranged from 39-71%. In Uganda in particular rates improved over time. These results are displayed in Figures 1 and 2.

How well (actual): Overall, as shown in Figures 1 and 2 intervention of the mSCC was as planned. Of interest was the fact that some pause points had stronger uptake than others. In particular, post-delivery and pre-discharge pause points were less frequently completed. As reflected in the qualitative work reported in this paper, this may be because health workers felt less urgency around the checklist once deliveries were safely completed.
